# Development of an immersive fall management program for nursing students: A usability study

**DOI:** 10.1371/journal.pone.0357026

**Published:** 2026-08-26

**Authors:** Mi Ok Song, So Young Yun

**Affiliations:** 1 Department of Nursing, Mokpo National University, Muan, Republic of Korea; 2 Department of Nursing, Nambu University, Gwangju, Republic of Korea; New York University Abu Dhabi, UNITED ARAB EMIRATES

## Abstract

This study aimed to develop an immersive virtual reality simulation–based fall management program (IVRsim-FMP) for nursing students, and to evaluate its usability and feasibility. IVRsim-FMP was developed following the System Development Life Cycle framework, and progressed through the analysis, design, implementation, and evaluation phases. The preliminary feasibility, system usability, cybersickness, and presence of the program were evaluated by 18 fourth-year nursing students. In the preliminary feasibility assessment, all the participants completed the study procedure. The system usability score was 77.50 (SD = 12.91), indicating an acceptable level; the total cybersickness score was 10.08 (SD = 9.53), suggesting a mild level; and the presence score was 150.94 (SD = 15.41), demonstrating a high level. IVRsim-FMP demonstrated acceptable usability and high immersion, indicating its potential as an educational tool in nursing education for fall prevention and management. This program provides opportunities to practice patient-safety scenarios that are difficult to replicate in clinical environments. The findings suggest that the IVRsim-FMP should be further explored as a training tool for fall prevention and management not only in nursing education but also in professional development for new and experienced nurses.

## Introduction

According to the World Health Organization (WHO) [1], millions of patients worldwide experience harm annually because of preventable errors in healthcare delivery. Institutional improvements and policy responses are essential to reduce patient safety incidents. In response, the WHO [2] introduced the Global Patient Safety Action Plan 2021–2030, outlining strategies to strengthen patient safety globally and encouraging countries to develop national policies. Consequently, improving the quality and safety of healthcare institutions has become a national priority in several countries.

Falls are among the most prevalent patient safety incidents and are defined as unexpected events in which a person descends to a level lower than their current position [3]. Falls comprise a significant proportion of medical accidents, necessitating continuous monitoring and preventive measures. Given that fall incidence is a global public health concern and a critical international patient safety goal [4], undergraduate nursing education must integrate comprehensive fall prevention and management strategy training.

Simulation-based learning is a pedagogical approach in nursing education that replicates real clinical environments using technological tools such as mannequins, videos, and role-playing techniques. This method enables students to practice specific procedures, make clinical decisions, and develop their critical thinking skills [5]. In recent years, virtual reality (VR) has gained prominence in simulation-based education as a modality that provides interactive, experiential, and repeatable learning opportunities in a safe environment [6–8], particularly for the current generations of students.

VR simulations employ various immersive, highly visual, and three-dimensional (3D) elements to replicate real-world scenarios and healthcare procedures [9]. Based on the level of immersion, VR simulations can be classified as non-immersive or immersive. Non-immersive VR simulations allow users to interact with a displayed environment through a computer monitor using input devices such as a mouse, keyboard, touchscreen, or joystick. In contrast, immersive virtual reality (IVR) simulations provide a fully simulated environment with multiple sensory output devices, including head-mounted displays, stereoscopic devices, audio systems, and haptic feedback tools [10]. IVR simulations enhance user engagement by providing sensory stimuli that correspond to the virtual environment, thereby fostering a heightened sense of presence within the simulation.

IVR simulations have shown to increase student engagement in simulated clinical environments, facilitate comprehension of nursing concepts, and enhance critical thinking skills for problem-solving in nursing education. They also enable students to practice nursing techniques in a controlled, risk-free environment without compromising patient safety [11–15].

Several studies have explored the integration of IVR simulation into nursing education, particularly in fundamental nursing courses focusing on procedural skills such as intravenous catheter insertion, nasogastric tube feeding, and personal protective equipment usage [11,12]. In addition, IVR simulation has been implemented in perioperative nursing practicum for undergraduate nursing students, as well as in pediatric nursing education, including neonatal assessment and resuscitation training [13–15].

Beyond technical and procedural skill development, IVR simulations have been employed to enhance non-technical nursing competencies. For example, in psychiatric nursing, it has been used to improve problem-solving skills when managing patients with various psychiatric conditions [16], and in adult nursing to enhance empathy in patient care [17]. A recent meta-analysis on the effectiveness of VR simulation in nursing education indicated that IVR simulation is more effective than traditional teaching methods in improving knowledge acquisition, clinical performance, satisfaction, and self-efficacy [6,7]. Furthermore, IVR simulation has been recognized as a cost-effective alternative to high-fidelity simulation, offering substantial educational benefits while reducing the resource burden [18].

Given these findings, the integration of an IVR simulation–based fall management program (IVRsim-FMP) may provide nursing students with opportunities to engage in fall prevention and management scenarios in a controlled, risk-free learning environment. Implementing innovative educational programs that address key patient safety topics is imperative for equipping nursing students with essential patient safety knowledge and fall prevention skills [19,20]. Although previous studies have explored VR application in patient safety education [8], to our knowledge, limited research has examined IVR simulation-based education specifically focusing on fall prevention and management for nursing students. Therefore, this study aimed to develop an IVRsim-FMP for nursing students and evaluate its feasibility and usability.

### Objectives

The primary objective of this study was to develop a VR simulation that reflects the clinical environment, incorporating fall prevention, post-fall patient assessment, and nursing interventions. In addition, this study aimed to evaluate the program’s preliminary feasibility, system usability, cybersickness, and presence for overall usability.

## Methods

### Study design

This study employed a methodological research design to develop and evaluate an IVR simulation program for fall prevention in nursing education. The study protocol was prospectively registered in the Open Science Framework (registration no. https://doi.org/10.17605/OSF.IO/ZPKU8).

### Participants

This study was a usability study using a pilot convenience sample. The study sample consisted of 18 fourth-year nursing students enrolled at N University in Gwangju, Republic of Korea. Recruitment was conducted through notices on the department bulletin board and online social media platforms, detailing the purpose of participation and its voluntary nature. The recruitment materials explicitly stated that participation was not mandatory and that non-participation would not result in any disadvantages.

Data were collected between May 12 and July 14, 2023. According to Lewis et al. [21], for an observational feasibility and usability study, a sample size of 18–25 participants is generally considered sufficient. Therefore, this study initially established a target sample size of 20 participants, accounting for a potential dropout rate of 10%. However, recruitment was concluded upon reaching the minimum required number of 18 participants as all enrolled participants provided complete responses and no attrition occurred during the study period. One participant reported visual discomfort because they had to remove their glasses while using the VR headset, but did not report any other symptoms and was able to complete the program.

### Instruments

#### Preliminary feasibility.

Preliminary feasibility was evaluated using the following indicators adapted from the criteria proposed by Kelleher et al. [22].

1. Successful recruitment and execution of the study over six months
2. Adherence to the experimental protocol in at least 80% of the cases
3. Data collection from at least 80% of the participants

#### Usability.

Using standardized tools, the usability was assessed through a post-intervention survey that measured system usability, cybersickness, and presence.

(1) System usability

The System Usability Scale (SUS) [23] was employed to evaluate the usability of the system. This scale subjectively assesses the usability of user interfaces and consists of ten items rated on a 5-point Likert scale ranging from 1 (“Strongly disagree”) to 5 (“Strongly agree”), including both positively and negatively worded items. The total score ranges from 0 to 100, with higher scores indicating greater system usability. In the original study, the tool demonstrated good reliability with Cronbach’s alpha = 0.85, whereas in this study, it was 0.795.

(2) Cybersickness

To assess the degree of cybersickness experienced in the VR environment, the Virtual Reality Sickness Questionnaire (VRSQ) [24], based on the Simulator Sickness Questionnaire (SSQ) [25], was used. The VRSQ consists of nine items across two subscales: Oculomotor Symptoms (four items) and Disorientation Symptoms (five items). Each item is rated on a 4-point scale ranging from 0 (“Not at all”) to 3 (“Very much”). The oculomotor score is calculated by dividing the sum of the four item scores by 12 and multiplying by 100, whereas the disorientation score is calculated by dividing the sum of the five item scores by 15 and multiplying by 100. The total VRSQ score is calculated as the mean of the two subscale scores. All scores range from 0 to 100, with higher scores indicating greater cybersickness. In the original study, Cronbach’s alpha was 0.847 for the oculomotor subscale and 0.886 for the disorientation subscale; Cronbach’s alpha for the total VRSQ was 0.633–0.674 in a subsequent validation study [26] and 0.657 in the present study.

(3) Presence

Presence was measured using the Presence Questionnaire (PQ) to assess the sense of presence and immersion in the simulation environment. The PQ was originally developed by Witmer and Singer [27] and later revised by Witmer et al. [28]. The scale consists of 24 items categorized into 7 subdomains: Realism (7 items), Possibility to Act (4 items), Quality of Interface (3 items), Possibility of Examine (3 items), Self-Evaluation of Performance (2 items), Sound (3 items), and Haptic Technology (2 items). Each item is rated on a 7-point Likert scale ranging from 1 (“Not at all”) to 7 (“Very much”), with total scores ranging from 24 to 168. Higher scores indicate a stronger sense of presence. The reliability of the original tool was Cronbach’s alpha = 0.88, whereas Witmer et al. [27] reported Cronbach’s alpha = 0.84. In this study, Cronbach’s alpha was 0.967.

Additionally, the start and end times of the simulation sessions were recorded for each participant, and the simulation completion time was calculated as the elapsed time between these two time points.

### Research procedure

#### Development of the IVRsim-FMP.

The IVRsim-FMP was developed following the System Development Life Cycle (SDLC) framework [29], comprising four phases: analysis, design, implementation, and evaluation.

(1) The analysis phase

In the analysis phase, the necessity of fall prevention education was established through a comprehensive review of the Korea Patient Safety Reporting and Learning System (KOPS) and relevant literature. Falls have a high incidence worldwide [1], and among patient safety incidents reported in the KOPS, falls are the most frequently occurring events [30]. Falls not only lead to physical injuries, but also have adverse social and psychological consequences, imposing a significant financial burden on healthcare systems [31–33]. Furthermore, strong evidence indicates that the incidence of falls decreases when patient education is implemented along with complementary training for healthcare professionals [34]. These findings underscore the need for fall prevention education for nursing students as future healthcare providers.

Previous studies on fall prevention programs for nurses have primarily utilized video-based and PowerPoint-supported training methodologies [35], individual and group education for both nurses and patients to enhance fall prevention efficacy [36], and patient simulators or standardized patients [37, 38]; however, the application of VR technology in this context, specifically targeting nursing students, has been limited. Given nurses’ crucial role in educating patients on fall prevention, the educational approach should actively engage current nursing students in fall prevention training.

(2) The design phase

In the design phase, the key content was defined based on the findings of the analysis phase, and the program was structured into Basic and Advanced levels. The Basic level focused on fall prevention nursing interventions for hospitalized patients, whereas the Advanced level addressed nursing interventions for managing fall incidents in hospitalized patients through case-based learning.

**Fig 1:**
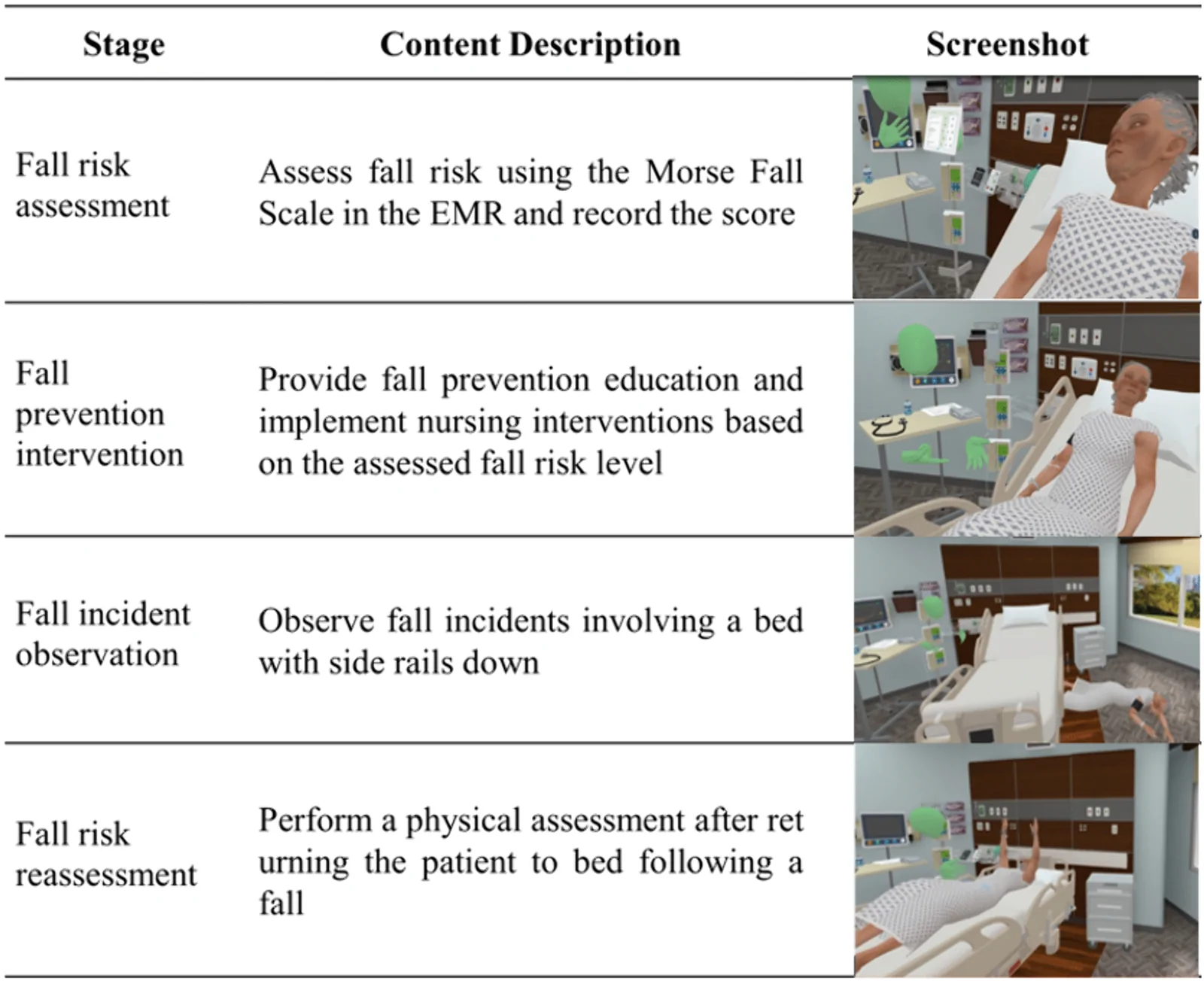
Snapshot of the IVRsim-FMP.

The program incorporated basic case overviews, patient demographics, medical histories, other relevant characteristics, environmental descriptions, and case progression scenarios to substantiate the case concept (Fig 1). Fig 1 shows a representative screenshot of the virtual ward environment. The nursing students interacted with patients and equipment in a simulated hospital setting to practice fall prevention and management scenarios. The program integrated immersive 3D graphics, task-based activities, and decision-making prompts to replicate real-world nursing contexts.

The development of the IVRsim-FMP was guided by Kolb’s experiential learning theory, which posits that learning is influenced by experiences involving thoughts, emotions, and environmental factors, and that learners acquire knowledge more effectively over four sequential learning cycles [39]. In addition, communication and interventions among healthcare professionals following fall incidents were structured according to the Situation, Background, Assessment, and Recommendation (SBAR) framework to ensure structured and effective clinical communication.

(3) The implementation phase

During the implementation phase, the designed content and algorithms were developed into an IVR program in collaboration with the VR simulation development company SimX (San Francisco, CA, USA). The development period was from July 2022 to January 2023.

The development team created a comprehensive user flow diagram based on the structured case specifications derived from the design phase, and mapped the learner’s journey through the simulation. The VR simulation incorporated the following elements:

- Electronic Medical Record (EMR) content with patient demographics and medical history
- X-ray films relevant to the clinical scenario
- Dialogue voiceovers tailored to each role in the simulation

In addition, upon completion of the program, the learners received a case report that enabled them to review their completion of actions over a timeline, to facilitate instructor assessments and learners’ reflections on their performance.

(4) The evaluation phase

In the evaluation phase, 18 nursing students participated in a systematic usability assessment of the content. The evaluation measured the system usability, cybersickness, and presence to determine the usability, feasibility, and potential applicability of the IVRsim-FMP in nursing education.

#### Operation of IVRsim-FMP.

(1) Orientation

The participants received a comprehensive orientation regarding the program operation, including information about the potential adverse effects that might occur during usage. They were specifically instructed to promptly notify a research assistant and discontinue participation upon experiencing two or more symptoms, such as visual or auditory disturbances, fatigue, or discomfort, during program utilization.

(2) Program execution

**Fig 2:**
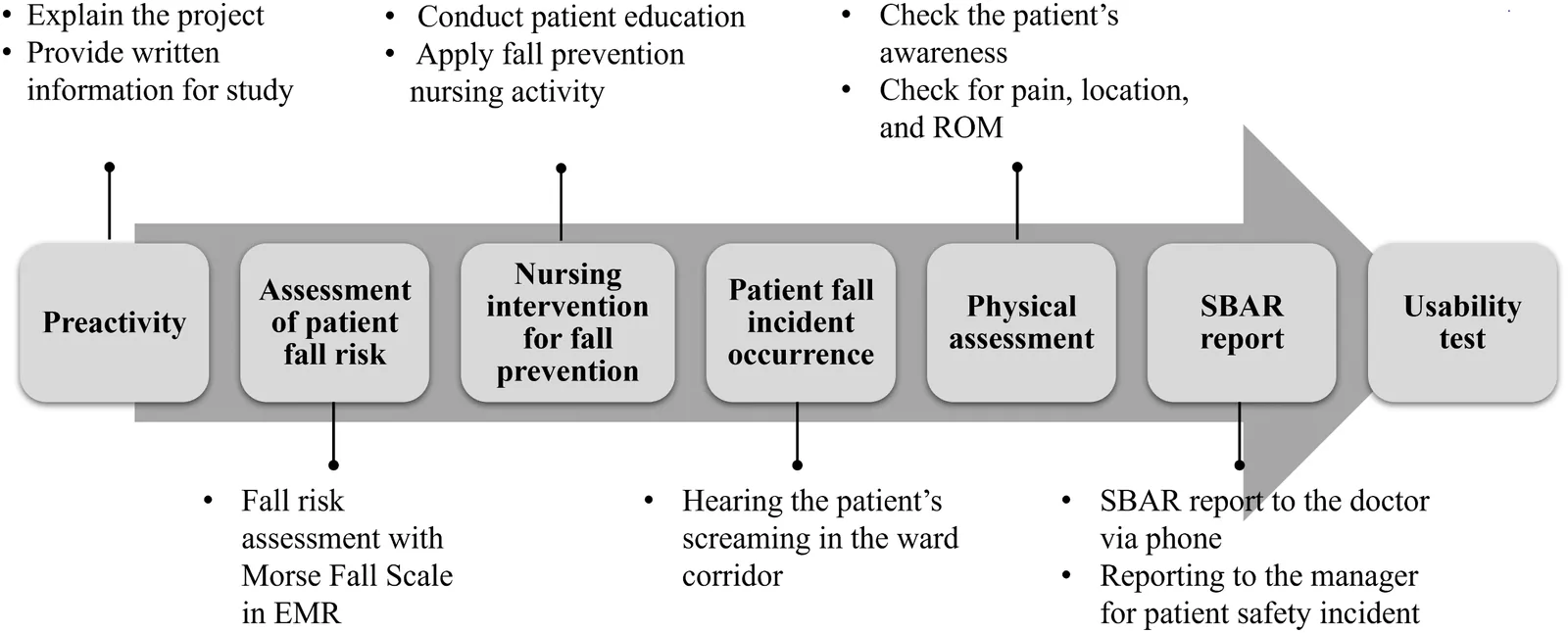
The procedural steps of the IVRsim-FMP program: orientation, scenario introduction, task performance, system feedback, and debriefing, designed based on the SDLC framework.

The procedural steps of the IVRsim-FMP program are illustrated in Fig 2, which includes the structured flow of the simulation, such as the orientation, scenario introduction, task performance, system feedback, and debriefing. Each step was designed according to the SDLC framework to ensure systematic implementation and educational relevance.

(3) Survey administration

Following the completion of the IVRsim-FMP session, the participants completed a survey assessing their general characteristics, system usability, cybersickness, and sense of presence.

### Data analysis methods

The collected data were analyzed using SPSS WIN 28.0. Participants’ general characteristics are summarized using frequencies and percentages, and the results of the measured variables are presented as means and standard deviations (SDs).

### Ethical considerations

This study was approved by the Institutional Review Board (IRB) of N University (Approval No.: 1041478–2023-HR-009) in the Republic of Korea. To ensure voluntary participation, the research assistants obtained informed consent from the participants before survey administration. The consent forms included assurances of anonymity and confidentiality. The participants who completed the study received compensation as gifts.

## Results

### General characteristics

Study participants were 18 fourth-year nursing students (men = 3 [16.7%], women = 15 [83.3%]; *M*_*age*_ = 22.5 years, SD = 1.86) (Table 1).

**Table 1:** Participants’ general characteristics (N = 18).

Variables	n (%)	M (SD)
**Age (year)**		22.5 (1.86)
**Gender**		
**Men**	3 (16.7)	
**Women**	15 (83.3)	
**Year Level**		
**Fourth Year**	18 (100)	

M = mean, SD = standard deviation

### Preliminary feasibility

The study was conducted from May 12 to July 14, 2023, and data collection was completed within the designated period. Preliminary feasibility was supported by 100% completion of the study procedures and data collection among the enrolled participants.

### Usability

#### System usability.

The mean system usability score was 77.50 (SD = 12.91), indicating an acceptable level of usability according to the usability criteria of GitLab [40] (Table 2).

**Table 2:** Results of the System Usability Scale (SUS) evaluation.

Item	Observed Range	M (SD)
1. I think that I would like to use this system frequently.	4–5	4.78 (0.43)
2. I found the system unnecessarily complex.	1–3	1.39 (0.61)
3. I thought the system was easy to use.	2–5	4.28 (0.83)
4. I think that I would need the support of a technical person to be able to use this system.	2–5	3.83 (0.99)
5. I found the various functions in this system were well integrated.	4–5	4.78 (0.43)
6. I thought there was too much inconsistency in this system.	1–4	1.50 (0.99)
7. I would imagine that most people would learn to use this system very quickly.	2–5	4.39 (0.92)
8. I found the system very cumbersome to use.	1–4	1.56 (0.86)
9. I felt very confident using the system.	2–5	4.44 (0.86)
10. I needed to learn a lot of things before I could get going with this system.	1–5	3.44 (1.38)
Total	45–95	77.50 (12.91)

M, mean; SD, standard deviation. Item-level scores are presented as raw scores on the original 1–5 agreement scale. The total SUS score was calculated using the standard SUS scoring method, in which negatively worded items were reverse-scored before conversion to a 0–100 scale.

#### Cybersickness.

Analysis of cybersickness revealed a VRSQ-Oculomotor score of 14.89 (SD = 15.05), a VRSQ-Disorientation score of 5.28 (SD = 6.69), and a VRSQ-Total score of 10.08 (SD = 9.53). These results indicate that the level of cybersickness experienced in the IVRsim-FMP group was mild (Table 3).

**Table 3:** Results of the virtual reality sickness questionnaire evaluation.

Item	Observed Range	M (SD)
1. General discomfort	0–2	0.33 (0.59)
2. Fatigue	0–2	0.28 (0.58)
3. Headache	0–1	0.06 (0.24)
4. Eyestrain	0–1	0.56 (0.51)
5. Difficulty focusing	0–2	0.61 (0.85)
6. Fullness of head	0–2	0.44 (0.62)
7. Blurred vision	0–2	0.22 (0.55)
8. Dizzy (eyes closed)	0	0 (0)
9. Vertigo	0–1	0.06 (0.24)
VRSQ-O	0–50	14.89 (15.05)
VRSQ-D	0–20	5.28 (6.69)
VRSQ-Total	0–31	10.08 (9.53)

M, mean; SD, standard deviation; VRSQ-O, Virtual Reality Sickness Questionnaire-Oculomotor; VRSQ-D, Virtual Reality Sickness Questionnaire-Disorientation

#### Sense of presence.

The overall mean score for presence was 150.94 (SD = 15.41). The mean scores for the individual questionnaire items are presented in Table 4.

**Table 4:** Results of the evaluation by subscale of the presence questionnaire.

Item	Observed Range	M (SD)
Realism	35–49	46.61 (3.60)
Possibility to Act	19–28	25.22 (2.94)
Quality of Interface	3–21	16.44 (5.46)
Possibility to Examine	15–21	19.05 (2.24)
Self-Evaluation of Performance	10–14	12.33 (1.53)
Sound	15–21	19.22 (2.37)
Haptic Technology	9–14	12.05 (2.10)
Total	114–168	150.94 (15.41)

M, mean; SD, standard deviation

#### Simulation completion time.

In addition, based on the recorded start and end times, the mean simulation completion time was 24.93 (SD = 8.80) min among the 18 participants with valid time records, with a range of 10–40 min. Excluding one participant who reported visual discomfort, the mean simulation completion time was 24.04 (SD = 7.26) min.

## Discussion

This study aimed to develop an IVR simulation program focusing on fall prevention, post-fall patient assessment, and nursing interventions as components of patient safety education. The program was designed to enable nursing students to assess fall risk in hospitalized patients and practice nursing interventions in clinical scenarios involving fall incidents. The evaluation of usability, cybersickness, and presence indicated that the program demonstrated acceptable usability and immersion, with manageable cybersickness symptoms.

To date, fall prevention education for nursing students has been implemented using fall management [37] and SBAR-based simulations [38]. However, to the best of our knowledge, limited research has applied clinical scenario–based VR simulations to fall prevention education for nursing students. Two previous studies [37, 38] are similar to the present study in that they addressed patient assessment, intervention, and post-fall evaluation as well as SBAR-based communication for fall management. Specifically, one study [37] incorporated both fall prevention education and incident-report documentation training. In contrast, the other study [38] integrated standardized patients into the practicum and implemented role exchange between participants to promote bidirectional learning. This approach differs from the immersive simulation model adopted in the present study, which allows repeated on-demand practice without the logistical constraints of mannequin- or Standardized Patient-based methods, potentially contributing to higher usability scores.

For effective fall prevention programs, interventions integrating environmental factors, education, nursing processes, and communication are essential [41]. The program developed in this study was designed to provide nursing students with opportunities to assess hospitalized patients’ fall risk using standardized tools and document EMR findings, aligning with nursing process principles, and address gaps identified in previous research. The realistic integration of environmental elements, such as visually witnessing a fallen patient on the hospital room floor and reviewing X-ray results within the virtual EMR, appears to have increased the program’s ecological and practical validity. The SBAR-based communication component was particularly significant, as it allowed nursing students to practice structured communication with healthcare professionals. Such experience is often difficult to obtain during clinical training. Because patient safety scenarios and clinical role-playing using mannequins or standardized patients have inherent limitations, this VR-based simulation appears to be a promising supplementary training tool.

VR has been demonstrated to facilitate learner development with greater empathy and an understanding of patients’ emotional needs [42, 43]. However, this study primarily focused on physical assessments and interventions for patients who have experienced a fall. Future research should enhance this program by incorporating emotional assessments and nursing interventions to support comprehensive patient-centered fall care. Moreover, integrating patient participation and emotional engagement strategies may further improve fall prevention education. Furthermore, in the context of patient safety education, the implementation of error-reporting mechanisms in a virtual environment warrants consideration.

Implementing innovative educational programs that address key patient safety topics is imperative for equipping nursing students with essential patient safety knowledge and fall prevention skills [19,20]. Although previous studies have explored VR application in patient safety education [8], research on IVR simulations is limited, particularly in the context of fall prevention.

The program achieved a high mean SUS score (M = 77.50, SD = 12.91), which fell within the “Good–Excellent” usability range according to GitLab criteria [40]. Compared to prior nursing education studies using VR or simulation-based systems, such as those of Kim (M = 74.40) [13] and Yu et al. (M = 67.59) [14], the results of this study indicate relatively higher perceived usability. This elevated usability score may be explained by the program’s intuitive interface design, simplified navigation pathways, and clearly structured task sequences, which collectively reduce the cognitive load and facilitate user engagement. In addition, the program yielded high overall presence scores (M = 150.94) with particularly elevated scores in the “realism” subdomain, which evaluates movement and immersion within the virtual space. This presence score was higher than that reported in a previous study that applied an IVR simulation to nursing students (M = 120.24) [44]. The higher presence observed in this study may be attributed to the integration of authentic clinical visuals, inclusion of virtual EMR functions for reviewing diagnostic results, and alignment of tasks with real-world nursing responsibilities, all of which enhance ecological validity. Given that immersion in VR simulations has been linked to improved learning outcomes [45], future research should examine whether the high presence achieved in this program is associated with measurable educational effects and further explore the potential influence of sociodemographic characteristics on these outcomes.

IVR simulation offers high levels of immersion and interactivity, making it a potentially useful educational tool for fall prevention. The findings of this study suggest that integrating IVR simulations into fall prevention education is a promising supplementary pedagogical approach and may provide students with opportunities to engage in patient safety scenarios in realistic and interactive learning environments. Such features may support the integration of IVR simulations as a supplementary approach to patient safety education [8, 46]. Future research should further investigate the educational effectiveness of IVRsim-FMP using objective learning outcomes such as knowledge, skill performance, and retention. Potential applications of this program beyond undergraduate nursing education, such as recurrent fall prevention and management training for newly graduated and experienced nurses, should be explored in future studies.

The inclusion of completion time data provides descriptive information regarding the participants’ interaction duration with the simulation scenario rather than educational effectiveness. In this study, the mean simulation completion time among the 18 participants was 24.93 (SD = 8.80) min, with a range of 10–40 min. Excluding one participant who reported visual discomfort, the mean completion time for the remaining participants was 24.04 (SD = 7.26) min. Simulation scenarios in nursing education are generally recommended to last between 15 and 30 min [44]. In prior VR-based nursing education studies, students engaged in a 20-min VR simulation scenario [47], and VR modules involving key nursing procedures required approximately 20–30 min to complete [48]. In another VR-based clinical procedure skills training study, each VR skill program was designed to last for 10 min, and a 10-min break was provided when VR practice exceeded 30 min to mitigate the potential for cybersickness [49]. Therefore, although the mean completion time in this study fell within the commonly reported range, the upper end of the observed range suggests that future versions of IVRsim-FMP should consider a target session duration of approximately 20–30 min and include optional pauses or breaks when sessions exceed 30 min or when users experience visual fatigue or cybersickness symptoms. The wide variation in completion time may also reflect differences in student familiarity with VR interfaces, task execution, and physical adaptation to the VR environment, including their visual comfort while using a headset.

During the study, one participant with poor eyesight reported visual difficulties when removing their glasses while wearing the VR headset. This aligns with the findings of previous studies, which suggest that VR-induced cybersickness can be exacerbated by fixed focal distances in VR headsets, making it difficult for users to adjust their focus and potentially reducing immersion [45]. In addition, the cybersickness assessment results indicated that “Eye Strain” and “Difficulty Focusing” scored higher than other discomfort-related items. Saab et al. [43] reported that visual problems represent the most significant barrier to VR utilization among nursing students, suggesting that this limitation is a common design constraint across various VR-based educational tools. Some VR headsets allow users to wear corrective glasses with spacer accessories. However, the VR equipment used in the present study did not include such accessories, which may have contributed to the visual discomfort reported by some participants. Future studies should consider equipment that accommodates users requiring visual correction to improve accessibility and comfort. Such advancements can facilitate more inclusive participation in VR-based learning environments.

Moreover, the reliability coefficients for VRSQ were lower than that reported in the original development study (Cronbach’s α = .657). Because cybersickness is a critical factor in both the usability and safety of VR-based education, cybersickness-related findings should be interpreted with caution. The lower reliability observed in this study may be attributed to the small and homogeneous sample size, differences in the VR content and environment, and potential response bias. Future studies are recommended to revalidate the instrument using larger samples, and more robust measurement approaches are required to validate these results.

### Limitations

This study used a small sample size from a single institution, which may have limited the statistical power and generalizability of the findings. Therefore, future studies should recruit larger and more diverse cohorts of participants from multiple institutions. In addition, as this was a pilot usability and feasibility study, no educational outcomes, such as knowledge acquisition, skill performance, or behavioral change, were assessed; thus, the educational effectiveness of the program remains to be established. Although simulation completion time was available as a preliminary objective indicator, no accuracy-based task performance metrics (e.g., step completion, error frequency, or success rate) were collected or analyzed. Accordingly, the present findings should be interpreted as evidence of usability and perceived immersion, rather than evidence of accurate task execution or superiority over conventional training methods. Because immersion was assessed through self-reported surveys, there may have been discrepancies between the participants’ subjective perceptions and their actual experiences. Future studies should incorporate objective behavioral measurements along with self-reports to enhance the accuracy of usability assessments and comprehensively evaluate the program’s impact on learning outcomes. Participant characteristics related to prior VR experience, gaming familiarity, visual correction, and susceptibility to motion sickness were not investigated in this study. Future studies should investigate these variables to provide a more comprehensive understanding of the factors that influence VR usability and user experience.

## Conclusion

The IVRsim-FMP program demonstrated acceptable usability and high presence, with only mild cybersickness, providing preliminary evidence that it may be a usable and immersive simulation tool with the potential for nursing education in fall prevention and post-fall patient care. By allowing students to engage in realistic patient-safety scenarios that may be difficult to replicate in authentic clinical settings, this program offers a feasible approach to scenario-based learning. Future research should build on these findings by assessing the impact of the program on objective learning outcomes and refining its realism and usability through user-centered design enhancements and additional immersive features. Such developments may inform the future use of VR-based simulations as a supplementary approach for patient safety education.

## Supporting information

S1 Data Raw data collected from study participants. (XLSX)
