# Supplementary material for: Development of an immersive fall management program for nursing students: A usability study
Source: PLoS One. 2026 Aug 26;21(8):e0357026. doi: 10.1371/journal.pone.0357026 (PMC13513987; doi:10.1371/journal.pone.0357026)
Supplement: Supplementary file 1 [file pone.0357026.s002.docx]

**Reviewer Comments**

1. Is the manuscript technically sound, and do the data support the conclusions?

Yes, with minor caveats.

The manuscript describes a technically sound pilot study that follows a systematic and well-established framework (System Development Life Cycle) for the development and preliminary evaluation of an Immersive Virtual Reality Simulation–Based Fall Management Program (IVRsim-FMP). The research design is appropriate for a usability study, and the methods are clearly described, including the use of validated instruments (SUS, VRSQ, Presence Questionnaire) to assess key outcomes: system usability, cybersickness, and sense of presence.

The data collection procedures were rigorous, and the sample size (n=18) is consistent with recommendations for pilot and usability studies in human-computer interaction research (e.g., Lewis et al., 2021, cited by the authors). All participants completed the study, and the data are presented clearly with appropriate descriptive statistics (means, standard deviations).

The conclusions regarding acceptable usability (SUS = 77.50) , mild cybersickness (VRSQ-Total = 9.89) , and high presence (PQ = 150.49) are directly supported by the data. The statistical analyses (descriptive statistics) are suitable for the study design and objectives.

However, the main caveat is that this is a usability and feasibility study, not an efficacy trial . The authors conclude that IVRsim-FMP is an "effective tool for educating nursing students," but the study did not measure educational outcomes such as knowledge gain, skill acquisition, or behavioral change. Therefore, while the program appears usable and immersive, its *educational effectiveness* remains unproven by the current data.

To improve accuracy, the authors should revise the conclusion to clarify that the program is a usable and immersive tool with potential for education , rather than asserting its educational effectiveness without empirical support from learning outcome data.

In summary, the study is methodologically sound for its purpose (usability evaluation), and the reported data support the conclusions about usability, presence, and cybersickness. The interpretation of "effectiveness" should be nuanced to reflect the pilot nature of the study and the absence of learning outcome measures.

*2. Has the statistical analysis been performed appropriately and rigorously?

Yes.

The statistical analysis in this study is appropriate and rigorously conducted for the aims of a pilot usability study. The authors employed descriptive statistics (frequencies, percentages, means, and standard deviations) to summarize the participants' demographic characteristics and the outcomes of interest, which is the correct approach given the study's exploratory and non-comparative design.

The data collection tools—System Usability Scale (SUS), Virtual Reality Sickness Questionnaire (VRSQ), and Presence Questionnaire (PQ)—are well-established and validated instruments, and their use is justified. The authors report the reliability of each instrument using Cronbach’s alpha , which is essential for assessing internal consistency. While the Cronbach’s alpha for the VRSQ (0.657) is slightly below the conventional threshold of 0.70, the authors acknowledge this in the limitations section, noting that it may reflect the tool's sensitivity in a small sample. This transparency strengthens the rigor of the reporting.

The analysis was conducted using SPSS 28.0 , a standard and reliable statistical software. The reporting of results is clear and consistent: means and standard deviations are appropriately presented for all key outcomes (e.g., SUS = 77.50 ± 12.91, PQ = 150.49 ± 15.4), and the data in tables align with the text (with the exception of a minor discrepancy in the total presence score—see major comment below).

Given that this is a usability and feasibility study without a control group or hypothesis testing, inferential statistics (e.g., t-tests, ANOVA) were not required. The focus on descriptive analysis is therefore methodologically sound.

Minor Note: There is a slight inconsistency between the text and Table 4: the manuscript reports a mean presence score of 150.49 , but Table 4 lists the total score as 150.94 . The authors should verify and correct this discrepancy for accuracy.

In summary, the statistical methods are well-suited to the research design, the analysis is correctly executed, and the results are transparently reported. No major statistical flaws were identified.

 3.Have the authors made all data underlying the findings in their manuscript fully available?

 Yes.

The authors have confirmed that all data underlying the findings described in the manuscript are fully available without restriction. In the "Data Availability" section of the submission, they state: *"All relevant data are within the manuscript and its Supporting Information files."*

This statement is consistent with PLOS ONE’s data policy. The manuscript includes descriptive statistics (means, standard deviations, frequencies, and percentages) for all key variables (e.g., SUS, VRSQ, PQ scores), and individual-level raw data or summary data sufficient to reproduce the results are expected to be included in the supporting files.

While the raw dataset (e.g., individual participant responses) is not visible in the current manuscript draft, the authors have indicated its presence in the Supporting Information, which is acceptable per PLOS policy. As a usability study with a small sample size (n=18), the full dataset should be readily shareable without compromising participant anonymity, especially since the ethical statement confirms that data were collected anonymously and with informed consent.

No restrictions on data sharing (e.g., third-party ownership or privacy concerns) are reported, and the authors have not cited any exceptional circumstances that would justify withholding data.

Therefore, the authors have complied with PLOS’s data availability requirements.

*4. Is the manuscript presented in an intelligible fashion and written in standard English?

Yes.

The manuscript is presented in a clear, logical, and intelligible manner, and it is written in fluent, standard English. The structure follows a conventional scientific format (Introduction, Methods, Results, Discussion), and the content flows coherently from one section to the next. The title, abstract, and keywords accurately reflect the study’s purpose and findings.

The language is formal, precise, and largely free of grammatical or typographical errors. Technical terms such as *immersive virtual reality simulation* , *system usability* , *cybersickness* , and *presence* are used appropriately and defined where necessary. The figures (Fig 1 and Fig 2) are relevant and support the understanding of the program design and procedural steps.

There are only a few minor linguistic issues that could be improved for clarity and consistency:

1. Line 181 : "hospitalized pa tients" appears to have a line-break error ("pa" and "tients" separated). This should be corrected to "hospitalized patients".
2. Line 270, Table 4 : The total mean presence score is reported as 150.49 in the text but 150.94 in the table. This discrepancy should be verified and corrected for accuracy.
3. Line 25 : "This program provides opportunities..." — consider changing "provides" to "provided" for tense consistency, as the rest of the abstract reports past-tense findings.

These are minor issues and do not hinder understanding. With these small corrections, the manuscript will meet the high linguistic standards expected by PLOS ONE.

In summary, the manuscript is well-written, intelligible, and suitable for publication after minor language polishing.

*5. Review Comments to the Author

This manuscript presents a timely and innovative study on the development and usability evaluation of an Immersive Virtual Reality Simulation–Based Fall Management Program (IVRsim-FMP) for nursing students. The integration of immersive virtual reality (IVR) into nursing education, particularly for a critical patient safety issue like fall prevention and management, holds significant promise. The study is well-conceived, systematically developed using the System Development Life Cycle (SDLC) framework, and rigorously evaluated for preliminary validity, usability, cybersickness, and presence .

The research design is appropriate for a pilot usability study, and the authors have employed validated instruments (SUS, VRSQ, Presence Questionnaire) to assess key outcomes. The findings—acceptable usability (SUS = 77.50), mild cybersickness (VRSQ-Total = 9.89), and high presence (PQ = 150.49)—are clearly reported and support the conclusion that IVRsim-FMP is a usable and immersive tool for nursing education.

The manuscript is well-written, logically structured, and adheres to PLOS ONE’s formatting and language standards. The data are fully available, ethical approval has been obtained, and there are no reported competing interests. Funding sources are clearly disclosed, and the statistical analysis is appropriate for the study’s objectives.

However, several points should be addressed to improve clarity, accuracy, and scientific rigor:

1. Clarify the Pilot Nature of the Study:

While the study is methodologically sound, the authors claim that IVRsim-FMP is an "effective tool for educating nursing students." This phrasing implies educational efficacy, but no learning outcomes (e.g., knowledge, skills, behavior change) were measured . The study evaluates *usability* , not *effectiveness* .

2. → Recommendation: Revise the conclusion to state that the program is a "usable and immersive tool with potential for education" rather than asserting its educational effectiveness. Emphasize that this is a feasibility/usability study , and future research should assess learning gains.
3. Inconsistency in Presence Score Reporting:

There is a discrepancy between the text and Table 4:

- Text reports mean presence score as 150.49 (line 268)
- Table 4 reports total score as 150.94

Please verify and correct this inconsistency for accuracy.

4. Reliability of the VRSQ:
5. The Cronbach’s alpha for the VRSQ is 0.657 , which is below the conventional threshold of 0.70 for acceptable internal consistency. While this is acknowledged in the limitations, it should be discussed more explicitly, as cybersickness is a critical factor in VR usability and user safety.
6. Minor Language and Formatting Issues:
   - Line 181: "hospitalized pa tients" appears to be a line-break error. Please correct to "hospitalized patients."
   - Line 25 (Abstract): "This program provides..." — consider changing to "provided" for tense consistency.
   - Ensure all figure captions are complete and descriptive in the final submission.
