# Supplementary material for: Development of an immersive fall management program for nursing students: A usability study
Source: PLoS One. 2026 Aug 26;21(8):e0357026. doi: 10.1371/journal.pone.0357026 (PMC13513987; doi:10.1371/journal.pone.0357026)
Supplement: Supplementary file 2 [file pone.0357026.s005.docx]

Dear Academic Editor and Reviewers,

We sincerely thank you for your careful review of our manuscript and for your valuable comments and suggestions. We have revised the manuscript accordingly and believe that these changes have improved the clarity and quality of the paper. Below, we provide a point-by-point response to each comment. All corresponding revisions have been incorporated into the revised manuscript.

Reviewer #2

Comment:
Please ensure consistency in the use of abbreviations throughout the text. Instances of inconsistent use of uppercase and lowercase letters, as well as repeated definition of the same abbreviation for a single term, were observed.

Response:
Thank you for your careful review and for pointing out this issue. In response to your comment, we thoroughly reviewed the manuscript and revised it to ensure consistency in abbreviations and terminology throughout the text and tables. Specifically, we corrected inconsistencies in uppercase/lowercase usage, standardized repeated terms and subscale labels, and removed redundant or repeated abbreviation definitions where applicable. These revisions were made throughout the manuscript to improve clarity and consistency (Lines 49-64, 222-242, 276-286).

Reviewer #3

Comment:
The general thesis of the paper is well formulated and the protocol and discussion is sound. However, there are some gaps that need addressing that I have included in my comments, which are attached. Most prominent among those is a deeper discussion about the absence of task performance metrics (on l.385) - is that data available? Even task completion times or success rates per participant would be a valuable metric of whether participants understood and were able to perform the task appropriately. It would also be valuable data for contrasting with the analogue version of the same nursing tasks and a justification of the claims the authors make around VR's superiority as a training tool. Please also include links to the figures, as I was unable to find them in the manuscript or reviewer portal.

Response:
We thank the reviewer for this insightful comment. We agree that the absence of objective task performance metrics was an important limitation of the original manuscript. In response, we re-examined our collected records and added simulation completion time to the Results section as a preliminary objective indicator of task execution. The mean completion time was 24.93 ± 8.80 minutes, with a range of 10 to 40 minutes (Lines 288–291).

We also revised the Discussion to provide a more in-depth interpretation of this finding. Specifically, we explain that the wide variation in completion times may reflect differences in technical proficiency and adaptation to the VR environment. We further added that some participants with poor eyesight experienced difficulty focusing and visual fatigue when using the headset without glasses, which may have interfered with rapid task performance (Lines 388–398).

In addition, we clarified in the Discussion and Limitations sections that, although completion time offers a preliminary objective measure, accuracy-based task performance indicators such as success rate, step completion, or error frequency were not collected in this study. Therefore, the present findings should be interpreted mainly as evidence of usability and perceived immersion rather than definitive evidence of task accuracy or superiority over analogue training approaches (Lines 388–398, 406–410).

Finally, we rechecked the figure files and confirmed that all figures (Fig. 1 and Fig. 2) are properly included in the revised submission.
