# Supplementary material for: Development of an immersive fall management program for nursing students: A usability study
Source: PLoS One. 2026 Aug 26;21(8):e0357026. doi: 10.1371/journal.pone.0357026 (PMC13513987; doi:10.1371/journal.pone.0357026)
Supplement: Supplementary file 3 [file pone.0357026.s006.docx]

| **Editor’s Comments** |  |
| --- | --- |
| 1. The intro has a clear rationale but there are still a few weaknesses as some language is awkward or grammatically off (please proof read). The "digital natives" argument is dated and I would recommend either removing or softening the sentence, or update. Also, the novelty claim that no prior research has investigated VR for fall prevention education in nursing students should be phrased more cautiously, for example "to our knowledge". The term "usefulness" in the abstract is broader than what was actually measured and "usability and feasibility" would be more precise. | Thank you for this helpful comment. We revised the Abstract and Introduction to improve clarity, grammar, and precision. In the Abstract, we replaced the term “usefulness” with “usability and feasibility” to more accurately reflect the outcomes measured in this study (line 16-18). We also removed the dated “digital natives” rationale and revised the sentence to focus on the educational affordances of VR, including interactive, experiential, and repeatable learning opportunities in a safe environment (line 48-51). In addition, we softened the novelty claim by adding “to our knowledge” and avoiding an absolute statement regarding previous research (line 87). We also proofread the Introduction and corrected awkward or ungrammatical wording. |
| 2. The SUS item reporting looks internally inconsistent. In Table 2, several negatively worded SUS items have very high means, for example: "I found the system unnecessarily complex" = 4.61, and "I thought there was too much inconsistency in this system" = 4.50. With the stated 1–5 agreement scale, those raw means would imply users agreed the system was complex/inconsistent/cumbersome, which conflicts with the reported total SUS score of 77.50 and the conclusion that usability was acceptable. This needs correction or explanation. Please justify. | Thank you for this important comment. We reviewed the SUS scoring and confirmed that the total SUS score of 77.50 was correctly calculated using the standard SUS scoring method. However, the previous version of Table 2 presented the reverse-scored values for negatively worded items while retaining the original negative item wording, which could lead to confusion. We revised Table 2 to present item-level scores as raw scores on the original 1–5 agreement scale. The total SUS score was retained, and a table note was added to clarify that negatively worded items were reverse-scored only for calculating the total SUS score on the 0–100 scale. |
| 3. The manuscript defines "preliminary validity" using recruitment, protocol adherence, and data completion criteria from a pilot/feasibility paper. Those are really feasibility/process indicators, not validity in the psychometric or methodological sense. I would advise renaming this section to something like feasibility indicators or preliminary feasibility throughout the abstract, methods, and results. | Thank you for this helpful comment. We agree that the indicators previously described as “preliminary validity,” including recruitment, protocol adherence, and data completion, are more appropriately interpreted as feasibility or process indicators rather than validity indicators in a psychometric or methodological sense. Accordingly, we revised the terminology throughout the Abstract, Methods, Results, and Discussion. Specifically, “preliminary validity” was replaced with “preliminary feasibility,” and the related criteria were described as feasibility indicators. We also revised the Results section to state that preliminary feasibility was supported by completion of the study procedures and data collection among enrolled participants (line 260). |
| 4. Several design details are missing: a. There are no participant characteristics related to prior VR experience, gaming familiarity, visual correction, or susceptibility to motion sickness, even though these are directly relevant to usability and cybersickness. b. The sample-size rationale is lightweight and ends with recruitment stopping at 18 rather than the initially planned 20. That is not fatal, but it should be described more cleanly as a pilot convenience sample. c. The methods say completion time was calculated among participants with valid time records, but the Results do not state how many participants had valid time data. d. The paper says participants should stop if they experienced two or more symptoms, but it does not explicitly state whether any participants paused, stopped early, or needed assistance. | Thank you for this helpful comment. The manuscript has been revised accordingly to reflect the reviewer’s comments.  a. Participant characteristics related to prior VR experience, gaming familiarity, visual correction, and susceptibility to motion sickness were not investigated in the present study. We acknowledge that these variables may influence usability perceptions and cybersickness experiences in VR environments. This limitation has been added to the limitation section: (line 436-439) “Participant characteristics related to prior VR experience, gaming familiarity, visual correction, and susceptibility to motion sickness were not investigated in this study. Future studies are needed to investigate these variables in order to provide a more comprehensive understanding of the factors influencing VR usability and user experience.”  b. The following sentence has been added to the Participants section: “This study was a usability study using a pilot convenience sample.” (line 104)  c. It was stated in the “Simulation Completion Time” section of the results that 18 participants had valid time records. (line 299)  d. The following content is described in the participant section of the methods: “One participant reported visual discomfort because they had to remove their glasses while using the VR headset; however, he did not report any other symptoms and were able to complete the program to the end”. (line 116-117) |
| 5. The descriptive results themselves are fine for a pilot usability paper, but a few parts need more details: a. Table ranges appear to be observed ranges, not instrument ranges, and that should be labeled clearly. b. The VRSQ reliability in this sample was low (a = 0.657), so conclusions about cybersickness should remain cautious. c. Completion time should not be treated as evidence of adequate task performance. d. The manuscript should avoid implying that high presence/usability means the program is educationally effective. | Thank you for this helpful comment. The manuscript has been revised accordingly to reflect the reviewer’s comments. "  a. The labels in Tables 2–4 was revised to “Observed Range.”  b. The following content was revised in the Discussion section.: the cybersickness-related findings should be interpreted with caution…. Future studies are recommended to revalidate the instrument using larger samples and more robust measurement approaches are needed to validate these results. (line 411-417)  c. The discussion regarding completion time was revised as follows.: The inclusion of completion-time data provides descriptive information regarding participants’ interaction duration with the simulation scenario, rather than educational effectiveness. (line 379-381)  d. To clearly indicate that the findings of this study were not intended to evaluate or establish the educational effectiveness of the program, we added the following statement to the Limitations section.: “as this was a pilot usability and feasibility study, no educational outcomes—such as knowledge acquisition, skill performance, or behavioral change—were assessed, and thus the educational effectiveness of the program remains to be established.” (line 422-425) |
| 6. Your discussion sometimes slides from "usable and immersive" into claims that the program supports patient-safety skill acquisition, transfer to clinical contexts, and broader professional training. Those claims may be plausible, but this study did not measure knowledge gain, skill accuracy, retention, comparison with standard teaching, or patient-safety outcomes. The current limitations section partly corrects this, but the discussion and conclusion should be brought into tighter alignment with the actual data. | Thank you for this comment. We revised the Discussion and Conclusion to avoid overstatement and to align the interpretation with the study outcomes. Claims related to patient-safety skill acquisition, clinical transfer, and broader professional training were softened, and we clarified that the educational effectiveness of IVRsim-FMP should be examined in future studies using objective learning outcomes. (line 374-378) |
| **Reviewers' comments:** |  |
| line 290 Simulation completion time section: it would be beneficial to have a projected ideal completion time as a comparison point - how long is the fastest possible playthrough, for example (presumably done by one of the authors, who designed the system?) Some discussion of the ideal VR session time for a training simulation (also included in the literature review) would be appropriate in relation to the discussion of visual fatigue on l 379. It would also flesh out this new section, which is only a single sentence long. | Thank you for this helpful suggestion. Because this study was designed to develop IVRsim-FMP and evaluate its preliminary feasibility and usability, a projected ideal completion time or fastest possible playthrough time was not predefined during program development. We therefore did not retrospectively estimate an ideal completion time. Instead, we expanded the Discussion by adding literature-based comparison points regarding recommended and reported VR simulation session durations. Specifically, we added evidence indicating that nursing simulation scenarios are generally recommended to last between 15 and 30 minutes and compared our mean completion time of 24.93 minutes with session durations reported in previous VR-based nursing education studies. We also added a discussion suggesting that future versions of IVRsim-FMP should consider a target session duration of approximately 20–30 minutes and include optional pauses or breaks when sessions exceed 30 minutes or when users experience visual fatigue or cybersickness symptoms (line 379-387). In addition, we linked the wide variation in completion time to students’ familiarity with VR interfaces, task execution, physical adaptation to the VR environment, and visual comfort while using the headset. (line 387-396) |
| line 395 - how many students encountered issues with their eye sight as a result of removing their glasses?  It would be good to note the mean time without including the eyesight-impaired group, especially given its impact on the SSQ. In my experience headsets have spacers that allow users to accommodate wearing glasses with an HMD to overcome this issue. | Thank you for this helpful comment.  The following content has been added to the participant section of the research methods.  “One participant reported visual discomfort because they had to remove their glasses while using the VR headset; however, they did not report any other symptoms and were able to complete the program to the end.”(line 116-117)  To further examine the potential influence of visual discomfort on simulation performance, we calculated the mean completion time excluding one participant in “Simulation completion time” section: “Excluding one participant who reported visual discomfort, the mean simulation completion time was 24.04 ± 7.26 minutes.” (line 302-303)  The following content was added to the discussions: “Some VR headsets allow users to wear corrective glasses through the use of spacer accessories. However, the VR equipment used in this study did not include such accessories, which may have contributed to the visual discomfort reported by some participants. Future studies should consider equipment that accommodates users requiring visual correction to improve accessibility and user comfort.” (line 405-410) |
| line 398 and 409 - no need to repeat the very similar wording in adjacent sections, I would remove it from the discussion. | We removed the repeated statement from the Discussion and retained the related limitation in the Limitations section to avoid redundancy and improve readability. |
