# Supplementary material for: Development of an immersive fall management program for nursing students: A usability study
Source: PLoS One. 2026 Aug 26;21(8):e0357026. doi: 10.1371/journal.pone.0357026 (PMC13513987; doi:10.1371/journal.pone.0357026)
Supplement: Supplementary file 4 [file pone.0357026.s007.docx]

**Reviewer #3**

We sincerely thank the reviewer for carefully reviewing our revised manuscript and for identifying the remaining issues related to the description and reporting of the VRSQ scores. We re-examined the original data, coding, scoring procedures, and all values reported in the manuscript. Our point-by-point responses are provided below.

| **Editor’s Comments** | Response |
| --- | --- |
| On l. 140 you say the system is based on a scale of 0–4 (Severe)—my understanding from Kim's paper is that the scale has 4 points, starting at 0, meaning the highest score is 3, not 4. | Thank you for identifying this error. The reviewer is correct that each VRSQ item is rated on a four-point scale ranging from 0 (none) to 3 (severe), rather than from 0 to 4.  We confirmed that the questionnaire administered to the participants, the coding of the original data, and the item-level analyses were all correctly based on the 0–3 response scale. Therefore, the observed ranges, means, and standard deviations of the individual VRSQ items did not require revision. The error was limited to the description of the response scale in the Methods section.  We have corrected the relevant sentence as follows:  “Each item is rated on a 4-point scale ranging from 0 (“Not at all”) to 3 (“Very much”).”  This correction has been made in the revised manuscript (Lines 140–141). |
| The paper calculates VRSQ-Total as a raw sum of the 9 items (max 36) but applies a scaled/weighted formula to VRSQ-O (max 50) and VRSQ-D (max 20), resulting in the Total score being lower than the Oculomotor subscale score (9.89 vs 14.89). Again, my understanding was that VRSQ-Total is calculated as the average of the two subscale percentages so that all three metrics sit on the exact same scale (0–100%). | Thank you for this important comment. The reviewer is correct that the VRSQ-Total score should be calculated as the mean of the percentage scores for the Oculomotor and Disorientation subscales, so that all three scores are expressed on the same scale ranging from 0 to 100.  We re-examined the original dataset and scoring procedure. All nine individual items had been correctly coded on a four-point scale ranging from 0 to 3; therefore, the observed ranges, means, and standard deviations of the individual items remained unchanged. However, the scoring description in the previous manuscript was unclear and could be interpreted as indicating that the VRSQ-Total score was calculated as the raw sum of the nine items.  We therefore recalculated and verified the VRSQ scores using the scoring method of the original instrument. The Oculomotor score was calculated by dividing the sum of the four item scores by 12 and multiplying by 100, and the Disorientation score was calculated by dividing the sum of the five item scores by 15 and multiplying by 100. The VRSQ-Total score was then calculated as the mean of these two subscale scores. Thus, the Oculomotor, Disorientation, and total scores are all expressed on the same 0–100 scale.  Following this correction, the VRSQ-Total score was revised from 9.89 (SD = 9.54) to 10.08 (SD = 9.53), with an observed range of 0–31. We updated the corresponding value in the Abstract, Results section, and Table 3 to ensure consistency throughout the manuscript. We also revised the Methods section to describe the scoring procedure explicitly and accurately.  The revised text reads as follows:  “The Oculomotor score is calculated by dividing the sum of the four item scores by 12 and multiplying by 100, whereas the Disorientation score is calculated by dividing the sum of the five item scores by 15 and multiplying by 100. The total VRSQ score is calculated as the mean of the two subscale scores. All scores range from 0 to 100, with higher scores indicating greater cybersickness.”  These revisions have been made in the Methods and Results sections and in Tables 3 (Lines 141–145). |
| Small detail, but the sub-scale's correct name in Table 4 should be Possibility to Examine, not Possibility to Examination. | Thank you for noting this error. We have corrected “Possibility to Examination” to “Possibility to Examine” in Methods and Table 4. We also reviewed the entire manuscript and the accompanying tables to ensure that the name of this subscale is presented consistently throughout the revised manuscript. |

**Additional revision**

In addition to the revisions made in response to the reviewer’s comments, we added a validation study reporting the reliability of the total VRSQ score. The corresponding text and reference list were updated, and all subsequent in-text citation numbers were renumbered accordingly (Lines 147–148; Reference 26).

We also revised two VRSQ item labels in Table 3 to ensure consistency with the terminology used in the original instrument: “Fullness of the head” was changed to “Fullness of head,” and “Dizziness with eyes closed” was changed to “Dizzy (eyes closed).”
