# Supplementary material for: Development of an immersive fall management program for nursing students: A usability study
Source: PLoS One. 2026 Aug 26;21(8):e0357026. doi: 10.1371/journal.pone.0357026 (PMC13513987; doi:10.1371/journal.pone.0357026)
Supplement: Peer review history

# Peer review: Decision Letter 0

**DOI:** 10.1371/journal.pone.0357026.r001

**Authors:** Roohollah Abbasi Shureshjani

11 Aug 2025

Dear Dr. Yun,

Thank you for submitting your manuscript to PLOS ONE. After careful consideration, we feel that it has merit but does not fully meet PLOS ONE’s publication criteria as it currently stands. Therefore, we invite you to submit a revised version of the manuscript that addresses the points raised during the review process.

Please submit your revised manuscript by Sep 25 2025 11:59PM. If you will need more time than this to complete your revisions, please reply to this message or contact the journal office at plosone@plos.org. When you're ready to submit your revision, log on to https://www.editorialmanager.com/pone/ and select the 'Submissions Needing Revision' folder to locate your manuscript file.

- A rebuttal letter that responds to each point raised by the academic editor and reviewer(s). You should upload this letter as a separate file labeled 'Response to Reviewers'.
- A marked-up copy of your manuscript that highlights changes made to the original version. You should upload this as a separate file labeled 'Revised Manuscript with Track Changes'.
- An unmarked version of your revised paper without tracked changes. You should upload this as a separate file labeled 'Manuscript'.

If you would like to make changes to your financial disclosure, please include your updated statement in your cover letter. Guidelines for resubmitting your figure files are available below the reviewer comments at the end of this letter.

If applicable, we recommend that you deposit your laboratory protocols in protocols.io to enhance the reproducibility of your results. Protocols.io assigns your protocol its own identifier (DOI) so that it can be cited independently in the future. For instructions see: https://journals.plos.org/plosone/s/submission-guidelines#loc-laboratory-protocols. Additionally, PLOS ONE offers an option for publishing peer-reviewed Lab Protocol articles, which describe protocols hosted on protocols.io. Read more information on sharing protocols at https://plos.org/protocols?utm_medium=editorial-email&utm_source=authorletters&utm_campaign=protocols.

We look forward to receiving your revised manuscript.

Kind regards,

Roohollah Abbasi Shureshjani, Ph. D.

Academic Editor

PLOS ONE

Journal Requirements:

When submitting your revision, we need you to address these additional requirements.

1. Please ensure that your manuscript meets PLOS ONE's style requirements, including those for file naming. The PLOS ONE style templates can be found at

https://journals.plos.org/plosone/s/file?id=wjVg/PLOSOne_formatting_sample_main_body.pdf and

https://journals.plos.org/plosone/s/file?id=ba62/PLOSOne_formatting_sample_title_authors_affiliations.pdf.

2. Please provide additional details regarding participant consent. In the ethics statement in the Methods and online submission information, please ensure that you have specified (1) whether consent was informed and (2) what type you obtained (for instance, written or verbal, and if verbal, how it was documented and witnessed). If your study included minors, state whether you obtained consent from parents or guardians. If the need for consent was waived by the ethics committee, please include this information.

If you are reporting a retrospective study of medical records or archived samples, please ensure that you have discussed whether all data were fully anonymized before you accessed them and/or whether the IRB or ethics committee waived the requirement for informed consent. If patients provided informed written consent to have data from their medical records used in research, please include this information.

3. Thank you for stating in your Funding Statement:

“This study was supported by the National Research Foundation of Korea (2019R1G1A1099327) and by research funds from Nambu University in 2023.”

Please provide an amended statement that declares *all* the funding or sources of support (whether external or internal to your organization) received during this study, as detailed online in our guide for authors at http://journals.plos.org/plosone/s/submit-now.  Please also include the statement “There was no additional external funding received for this study.” in your updated Funding Statement.

Please include your amended Funding Statement within your cover letter. We will change the online submission form on your behalf.

4. Thank you for stating the following financial disclosure:

“This study was supported by the National Research Foundation of Korea (2019R1G1A1099327) and by research funds from Nambu University in 2023.”

Please state what role the funders took in the study.  If the funders had no role, please state: "The funders had no role in study design, data collection and analysis, decision to publish, or preparation of the manuscript."

If this statement is not correct you must amend it as needed.

Please include this amended Role of Funder statement in your cover letter; we will change the online submission form on your behalf.

5. We note that your Data Availability Statement is currently as follows: [All relevant data are within the manuscript and its Supporting Information files.]

Please confirm at this time whether or not your submission contains all raw data required to replicate the results of your study. Authors must share the “minimal data set” for their submission. PLOS defines the minimal data set to consist of the data required to replicate all study findings reported in the article, as well as related metadata and methods (https://journals.plos.org/plosone/s/data-availability#loc-minimal-data-set-definition).

For example, authors should submit the following data:

- The values behind the means, standard deviations and other measures reported;

- The values used to build graphs;

- The points extracted from images for analysis.

Authors do not need to submit their entire data set if only a portion of the data was used in the reported study.

If your submission does not contain these data, please either upload them as Supporting Information files or deposit them to a stable, public repository and provide us with the relevant URLs, DOIs, or accession numbers. For a list of recommended repositories, please see https://journals.plos.org/plosone/s/recommended-repositories.

If there are ethical or legal restrictions on sharing a de-identified data set, please explain them in detail (e.g., data contain potentially sensitive information, data are owned by a third-party organization, etc.) and who has imposed them (e.g., an ethics committee). Please also provide contact information for a data access committee, ethics committee, or other institutional body to which data requests may be sent. If data are owned by a third party, please indicate how others may request data access.

6. Thank you for stating the following in the Acknowledgments Section of your manuscript:

“This study was supported by the National Research Foundation of Korea (2019R1G1A1099327) and by research funds from Nambu University in 2023.”

We note that you have provided additional information within the Acknowledgements Section that is not currently declared in your Funding Statement. Please note that funding information should not appear in the Acknowledgments section or other areas of your manuscript. We will only publish funding information present in the Funding Statement section of the online submission form.

Please remove any funding-related text from the manuscript and let us know how you would like to update your Funding Statement. Currently, your Funding Statement reads as follows:

“This study was supported by the National Research Foundation of Korea (2019R1G1A1099327) and by research funds from Nambu University in 2023.”

Please include your amended statements within your cover letter; we will change the online submission form on your behalf.

7. If the reviewer comments include a recommendation to cite specific previously published works, please review and evaluate these publications to determine whether they are relevant and should be cited. There is no requirement to cite these works unless the editor has indicated otherwise.

[Note: HTML markup is below. Please do not edit.]

Reviewers' comments:

Reviewer's Responses to Questions

**Comments to the Author**

1. Is the manuscript technically sound, and do the data support the conclusions?

Reviewer #1: Yes

Reviewer #2: Yes

**********

2. Has the statistical analysis been performed appropriately and rigorously?

Reviewer #1: Yes

Reviewer #2: I Don't Know

**********

3. Have the authors made all data underlying the findings in their manuscript fully available?

The PLOS Data policy

Reviewer #1: Yes

Reviewer #2: Yes

**********

4. Is the manuscript presented in an intelligible fashion and written in standard English?

Reviewer #1: Yes

Reviewer #2: No

**********

Reviewer #1: This manuscript presents a timely and innovative study on the development and usability evaluation of an Immersive Virtual Reality Simulation–Based Fall Management Program (IVRsim-FMP) for nursing students. The integration of immersive virtual reality (IVR) into nursing education, particularly for a critical patient safety issue like fall prevention and management, holds significant promise. The study is well-conceived, systematically developed using the System Development Life Cycle (SDLC) framework, and rigorously evaluated for preliminary validity, usability, cybersickness, and presence .

The research design is appropriate for a pilot usability study, and the authors have employed validated instruments (SUS, VRSQ, Presence Questionnaire) to assess key outcomes. The findings—acceptable usability (SUS = 77.50), mild cybersickness (VRSQ-Total = 9.89), and high presence (PQ = 150.49)—are clearly reported and support the conclusion that IVRsim-FMP is a usable and immersive tool for nursing education.

The manuscript is well-written, logically structured, and adheres to PLOS ONE’s formatting and language standards. The data are fully available, ethical approval has been obtained, and there are no reported competing interests. Funding sources are clearly disclosed, and the statistical analysis is appropriate for the study’s objectives.

However, several points should be addressed to improve clarity, accuracy, and scientific rigor:

1. Clarify the Pilot Nature of the Study:

While the study is methodologically sound, the authors claim that IVRsim-FMP is an "effective tool for educating nursing students." This phrasing implies educational efficacy, but no learning outcomes (e.g., knowledge, skills, behavior change) were measured . The study evaluates usability , not effectiveness .

→ Recommendation: Revise the conclusion to state that the program is a "usable and immersive tool with potential for education" rather than asserting its educational effectiveness. Emphasize that this is a feasibility/usability study , and future research should assess learning gains.

2. Inconsistency in Presence Score Reporting:

There is a discrepancy between the text and Table 4:

• Text reports mean presence score as 150.49 (line 268)

• Table 4 reports total score as 150.94

Please verify and correct this inconsistency for accuracy.

3. Reliability of the VRSQ:

The Cronbach’s alpha for the VRSQ is 0.657 , which is below the conventional threshold of 0.70 for acceptable internal consistency. While this is acknowledged in the limitations, it should be discussed more explicitly, as cybersickness is a critical factor in VR usability and user safety.

4. Minor Language and Formatting Issues:

• Line 181: "hospitalized pa tients" appears to be a line-break error. Please correct to "hospitalized patients."

• Line 25 (Abstract): "This program provides..." — consider changing to "provided" for tense consistency.

• Ensure all figure captions are complete and descriptive in the final submission.

Reviewer Comments

1. Is the manuscript technically sound, and do the data support the conclusions?

Yes, with minor caveats.

The manuscript describes a technically sound pilot study that follows a systematic and well-established framework (System Development Life Cycle) for the development and preliminary evaluation of an Immersive Virtual Reality Simulation–Based Fall Management Program (IVRsim-FMP). The research design is appropriate for a usability study, and the methods are clearly described, including the use of validated instruments (SUS, VRSQ, Presence Questionnaire) to assess key outcomes: system usability, cybersickness, and sense of presence.

The data collection procedures were rigorous, and the sample size (n=18) is consistent with recommendations for pilot and usability studies in human-computer interaction research (e.g., Lewis et al., 2021, cited by the authors). All participants completed the study, and the data are presented clearly with appropriate descriptive statistics (means, standard deviations).

The conclusions regarding acceptable usability (SUS = 77.50) , mild cybersickness (VRSQ-Total = 9.89) , and high presence (PQ = 150.49) are directly supported by the data. The statistical analyses (descriptive statistics) are suitable for the study design and objectives.

However, the main caveat is that this is a usability and feasibility study, not an efficacy trial . The authors conclude that IVRsim-FMP is an "effective tool for educating nursing students," but the study did not measure educational outcomes such as knowledge gain, skill acquisition, or behavioral change. Therefore, while the program appears usable and immersive, its educational effectiveness remains unproven by the current data.

To improve accuracy, the authors should revise the conclusion to clarify that the program is a usable and immersive tool with potential for education , rather than asserting its educational effectiveness without empirical support from learning outcome data.

In summary, the study is methodologically sound for its purpose (usability evaluation), and the reported data support the conclusions about usability, presence, and cybersickness. The interpretation of "effectiveness" should be nuanced to reflect the pilot nature of the study and the absence of learning outcome measures.

*2. Has the statistical analysis been performed appropriately and rigorously?

Yes.

The statistical analysis in this study is appropriate and rigorously conducted for the aims of a pilot usability study. The authors employed descriptive statistics (frequencies, percentages, means, and standard deviations) to summarize the participants' demographic characteristics and the outcomes of interest, which is the correct approach given the study's exploratory and non-comparative design.

The data collection tools—System Usability Scale (SUS), Virtual Reality Sickness Questionnaire (VRSQ), and Presence Questionnaire (PQ)—are well-established and validated instruments, and their use is justified. The authors report the reliability of each instrument using Cronbach’s alpha , which is essential for assessing internal consistency. While the Cronbach’s alpha for the VRSQ (0.657) is slightly below the conventional threshold of 0.70, the authors acknowledge this in the limitations section, noting that it may reflect the tool's sensitivity in a small sample. This transparency strengthens the rigor of the reporting.

The analysis was conducted using SPSS 28.0 , a standard and reliable statistical software. The reporting of results is clear and consistent: means and standard deviations are appropriately presented for all key outcomes (e.g., SUS = 77.50 ± 12.91, PQ = 150.49 ± 15.4), and the data in tables align with the text (with the exception of a minor discrepancy in the total presence score—see major comment below).

Given that this is a usability and feasibility study without a control group or hypothesis testing, inferential statistics (e.g., t-tests, ANOVA) were not required. The focus on descriptive analysis is therefore methodologically sound.

Minor Note: There is a slight inconsistency between the text and Table 4: the manuscript reports a mean presence score of 150.49 , but Table 4 lists the total score as 150.94 . The authors should verify and correct this discrepancy for accuracy.

In summary, the statistical methods are well-suited to the research design, the analysis is correctly executed, and the results are transparently reported. No major statistical flaws were identified.

3.Have the authors made all data underlying the findings in their manuscript fully available?

Yes.

The authors have confirmed that all data underlying the findings described in the manuscript are fully available without restriction. In the "Data Availability" section of the submission, they state: "All relevant data are within the manuscript and its Supporting Information files."

This statement is consistent with PLOS ONE’s data policy. The manuscript includes descriptive statistics (means, standard deviations, frequencies, and percentages) for all key variables (e.g., SUS, VRSQ, PQ scores), and individual-level raw data or summary data sufficient to reproduce the results are expected to be included in the supporting files.

While the raw dataset (e.g., individual participant responses) is not visible in the current manuscript draft, the authors have indicated its presence in the Supporting Information, which is acceptable per PLOS policy. As a usability study with a small sample size (n=18), the full dataset should be readily shareable without compromising participant anonymity, especially since the ethical statement confirms that data were collected anonymously and with informed consent.

No restrictions on data sharing (e.g., third-party ownership or privacy concerns) are reported, and the authors have not cited any exceptional circumstances that would justify withholding data.

Therefore, the authors have complied with PLOS’s data availability requirements.

*4. Is the manuscript presented in an intelligible fashion and written in standard English?

Yes.

The manuscript is presented in a clear, logical, and intelligible manner, and it is written in fluent, standard English. The structure follows a conventional scientific format (Introduction, Methods, Results, Discussion), and the content flows coherently from one section to the next. The title, abstract, and keywords accurately reflect the study’s purpose and findings.

The language is formal, precise, and largely free of grammatical or typographical errors. Technical terms such as immersive virtual reality simulation , system usability , cybersickness , and presence are used appropriately and defined where necessary. The figures (Fig 1 and Fig 2) are relevant and support the understanding of the program design and procedural steps.

There are only a few minor linguistic issues that could be improved for clarity and consistency:

1. Line 181 : "hospitalized pa tients" appears to have a line-break error ("pa" and "tients" separated). This should be corrected to "hospitalized patients".

2. Line 270, Table 4 : The total mean presence score is reported as 150.49 in the text but 150.94 in the table. This discrepancy should be verified and corrected for accuracy.

3. Line 25 : "This program provides opportunities..." — consider changing "provides" to "provided" for tense consistency, as the rest of the abstract reports past-tense findings.

These are minor issues and do not hinder understanding. With these small corrections, the manuscript will meet the high linguistic standards expected by PLOS ONE.

In summary, the manuscript is well-written, intelligible, and suitable for publication after minor language polishing.

*5. Review Comments to the Author

This manuscript presents a timely and innovative study on the development and usability evaluation of an Immersive Virtual Reality Simulation–Based Fall Management Program (IVRsim-FMP) for nursing students. The integration of immersive virtual reality (IVR) into nursing education, particularly for a critical patient safety issue like fall prevention and management, holds significant promise. The study is well-conceived, systematically developed using the System Development Life Cycle (SDLC) framework, and rigorously evaluated for preliminary validity, usability, cybersickness, and presence .

The research design is appropriate for a pilot usability study, and the authors have employed validated instruments (SUS, VRSQ, Presence Questionnaire) to assess key outcomes. The findings—acceptable usability (SUS = 77.50), mild cybersickness (VRSQ-Total = 9.89), and high presence (PQ = 150.49)—are clearly reported and support the conclusion that IVRsim-FMP is a usable and immersive tool for nursing education.

The manuscript is well-written, logically structured, and adheres to PLOS ONE’s formatting and language standards. The data are fully available, ethical approval has been obtained, and there are no reported competing interests. Funding sources are clearly disclosed, and the statistical analysis is appropriate for the study’s objectives.

However, several points should be addressed to improve clarity, accuracy, and scientific rigor:

1. Clarify the Pilot Nature of the Study:

While the study is methodologically sound, the authors claim that IVRsim-FMP is an "effective tool for educating nursing students." This phrasing implies educational efficacy, but no learning outcomes (e.g., knowledge, skills, behavior change) were measured . The study evaluates usability , not effectiveness .

2. → Recommendation: Revise the conclusion to state that the program is a "usable and immersive tool with potential for education" rather than asserting its educational effectiveness. Emphasize that this is a feasibility/usability study , and future research should assess learning gains.

3. Inconsistency in Presence Score Reporting:

There is a discrepancy between the text and Table 4:

• Text reports mean presence score as 150.49 (line 268)

• Table 4 reports total score as 150.94

Please verify and correct this inconsistency for accuracy.

4. Reliability of the VRSQ:

5. The Cronbach’s alpha for the VRSQ is 0.657 , which is below the conventional threshold of 0.70 for acceptable internal consistency. While this is acknowledged in the limitations, it should be discussed more explicitly, as cybersickness is a critical factor in VR usability and user safety.

6. Minor Language and Formatting Issues:

• Line 181: "hospitalized pa tients" appears to be a line-break error. Please correct to "hospitalized patients."

• Line 25 (Abstract): "This program provides..." — consider changing to "provided" for tense consistency.

• Ensure all figure captions are complete and descriptive in the final submission.

Reviewer #2: Core Methodological Concerns:

1. Inadequate sample size

The limited cohort (n=18) of final-year nursing students from a single institution undermines statistical power and generalizability.

2. Absence of effectiveness evaluation

The study fails to assess educational outcomes (knowledge acquisition, skill transfer, or behavioral change) despite claiming the program is "effective."(describe)

3. Omission of psycho-social dimensions

Critical psychological and social aspects of fall management are neglected in scenario design.

4. Deficient data analysis and discussion

Weak interpretation of results and insufficient bench-marking against existing literature diminish the study’s scholarly rigor.

Technical and Presentation-al Issues:

5. Language quality deficiencies

The manuscript contains significant grammatical errors, inconsistent tense usage, and stylistic flaws requiring comprehensive editing by a native English speaker or professional language service.

6. Referencing inconsistencies

non-uniform citation formatting violates journal guidelines.

7. Figure resolution

Figures 1 and 2 are of low quality in the submitted PDF file.

**********

what does this mean?). If published, this will include your full peer review and any attached files.

If you choose “no”, your identity will remain anonymous but your review may still be made public.

**Do you want your identity to be public for this peer review?**  For information about this choice, including consent withdrawal, please see our Privacy Policy

Reviewer #1: No

Reviewer #2: No

**********

[NOTE: If reviewer comments were submitted as an attachment file, they will be attached to this email and accessible via the submission site. Please log into your account, locate the manuscript record, and check for the action link "View Attachments". If this link does not appear, there are no attachment files.]

While revising your submission, please upload your figure files to the Preflight Analysis and Conversion Engine (PACE) digital diagnostic tool, https://pacev2.apexcovantage.com/. PACE helps ensure that figures meet PLOS requirements. To use PACE, you must first register as a user. Registration is free. Then, login and navigate to the UPLOAD tab, where you will find detailed instructions on how to use the tool. If you encounter any issues or have any questions when using PACE, please email PLOS at figures@plos.org. Please note that Supporting Information files do not need this step.

Attachment Submitted filename: Reviewer Comments.docx

# Author comment: Author response to Decision Letter 1

**DOI:** 10.1371/journal.pone.0357026.r002

29 Sep 2025

September 15, 2025

Roohollah Abbasi Shureshjani, Ph. D.

Academic Editor

PLOS One

Dear Dr. Shuresjani:

We would like to thank you and the reviewers for your time and constructive feedback for the improvement of our manuscript entitled “Development of an immersive fall management program for nursing students: A usability study” (PONE-D-25-23302R1), which we submitted to PLOS One. As requested, we are including this response to the decision letter. Please see our responses to the reviewers’ comments at the bottom of this letter.

We hope that the changes we made have significantly improved the quality of our manuscript. Please do let us know if there is anything else we can do at this stage. We would be more than happy to do everything we can to assist you in this process.

We look forward to hearing from you!

Best regards,

So Young Yun

Associate Professor

Department of Nursing,

Nambu University,

1 Nambudae-gil, Gwangsan-gu,

Gwangju, 62271 Republic of Korea

Tel.: +82-62-970-0223

yunsy@nambu.ac.kr

Reviewers’ Comments

Number of Reviewers: 2

Reviewer 1

1. There are only a few minor linguistic issues that could be improved for clarity and consistency:

a. Line 181 : "hospitalized pa tients" appears to have a line-break error ("pa" and "tients" separated). This should be corrected to "hospitalized patients".

b. Line 270, Table 4 : The total mean presence score is reported as 150.49 in the text but 150.94 in the table. This discrepancy should be verified and corrected for accuracy.

c. Line 25 : "This program provides opportunities..." — consider changing "provides" to "provided" for tense consistency, as the rest of the abstract reports past-tense findings.

These are minor issues and do not hinder understanding. With these small corrections, the manuscript will meet the high linguistic standards expected by PLOS ONE.

In summary, the manuscript is well-written, intelligible, and suitable for publication after minor language polishing.

Response: Thank you for this feedback. Corrections were properly implemented to address the comment.

Change:

a. “The Basic level focuses on fall prevention nursing interventions for hospitalized patients” (Lines 184–185)

b. “The overall mean score for presence was 150.94 (SD = 15.41), with mean scores for the individual questionnaire items presented in Table 4.” (Lines 281–282)

Table 4. Results of the Evaluation by Subscale of the Presence Questionnaire

Item Range M (SD)

Realism 35–49 46.61 (3.60)

Possibility to act 19–28 25.22 (2.94)

Quality of the interface 3–21 16.44 (5.46)

Possibility to examine 15–21 19.05 (2.24)

Self-evaluation of performance 10–14 12.33 (1.53)

Sounds 15–21 19.22 (2.37)

Haptic 9–14 12.05 (2.10)

Total 114–168 150.94 (15.41)

c. “This program provided opportunities to practice patient safety scenarios that are difficult to replicate in clinical environments.” (Lines 23–24)

2. Clarify the Pilot Nature of the Study:

While the study is methodologically sound, the authors claim that IVRsim-FMP is an "effective tool for educating nursing students." This phrasing implies educational efficacy, but no learning outcomes (e.g., knowledge, skills, behavior change) were measured . The study evaluates usability , not effectiveness .

Response: Thank you for this feedback. Corrections were properly implemented to address the comment, and a native English speaker further edited the manuscript.

Change: “IVRsim-FMP demonstrated acceptable usability and high immersion, indicating potential as an educational tool for nursing education on fall prevention and management.” (Lines 21–23)

3. → Recommendation: Revise the conclusion to state that the program is a "usable and immersive tool with potential for education" rather than asserting its educational effectiveness. Emphasize that this is a feasibility/usability study , and future research should assess learning gains.

Response: Thank you for this feedback. Corrections were properly implemented to address the comment.

Change: “The IVRsim-FMP program demonstrated acceptable usability, mild cybersickness, and high presence, indicating that it is a usable and immersive simulation tool with potential for nursing education in fall prevention and postfall patient care.” (Lines 392–394)

4. Inconsistency in Presence Score Reporting:

There is a discrepancy between the text and Table 4:

a. Text reports mean presence score as 150.49 (line 268)

b. Table 4 reports total score as 150.94

Please verify and correct this inconsistency for accuracy.

Response: Thank you for this feedback. Corrections were properly implemented to address the comment.

Change: “The overall mean score for presence was 150.94 (SD = 15.41), with mean scores for the individual questionnaire items presented in Table 4.” (Lines 281–282)

Table 4. Results of the Evaluation by Subscale of the Presence Questionnaire

Item Range M (SD)

Realism 35–49 46.61 (3.60)

Possibility to act 19–28 25.22 (2.94)

Quality of the interface 3–21 16.44 (5.46)

Possibility to examine 15–21 19.05 (2.24)

Self-evaluation of performance 10–14 12.33 (1.53)

Sounds 15–21 19.22 (2.37)

Haptic 9–14 12.05 (2.10)

Total 114–168 150.94 (15.41)

5. Reliability of the VRSQ:

Response: It is understood that the relatively low internal consistency of the VRSQ (Cronbach’s alpha = 0.657) warrants a more explicit discussion, given that cybersickness directly affects VR usability and user safety. To address this, we have expanded the Discussion section to highlight possible reasons for the lower reliability observed in our study, such as the small and homogeneous sample, differences in VR content and environment, and potential response bias. We also emphasized that future research should revalidate the VRSQ in larger and more diverse cohorts to ensure consistent measurement quality.

Change: “Moreover, the reliability coefficients for VRSQ were lower than those reported in the original development study (α = .657). Since cybersickness is a critical factor for both the usability and safety of VR-based education, this limitation should be interpreted with caution. The lower reliability observed in this study may be attributable to the small and homogeneous sample, differences in VR content and environment, and potential response bias. Therefore, these results should be interpreted with caution, and future studies are recommended to revalidate the instrument across diverse samples and settings to ensure consistent measurement quality.” (Lines 372–379)

6. The Cronbach’s alpha for the VRSQ is 0.657, which is below the conventional threshold of 0.70 for acceptable internal consistency. While this is acknowledged in the limitations, it should be discussed more explicitly, as cybersickness is a critical factor in VR usability and user safety.

Response: Thank you for pointing out this important issue. We agree that the relatively low internal consistency of the VRSQ (Cronbach’s alpha = 0.657) warrants a more explicit discussion, given that cybersickness directly affects VR usability and user safety. To address this, we have expanded the Discussion section to highlight possible reasons for the lower reliability observed in our study, such as the small and homogeneous sample, differences in VR content and environment, and potential response bias. We also emphasized that future research should revalidate the VRSQ in larger and more diverse cohorts to ensure consistent measurement quality.

Change: “Moreover, the reliability coefficients for VRSQ were lower than those reported in the original development study (α = .657). Since cybersickness is a critical factor for both the usability and safety of VR-based education, this limitation should be interpreted with caution. The lower reliability observed in this study may be attributable to the small and homogeneous sample, differences in VR content and environment, and potential response bias. Therefore, these results should be interpreted with caution, and future studies are recommended to revalidate the instrument across diverse samples and settings to ensure consistent measurement quality.” (Lines 372–379)

7. Ensure all figure captions are complete and descriptive in the final submission.

Response: Upon review, the manuscript’s figure captions were ensured to be complete and to describe the figure’s content.

Reviewer 2

Core Methodological Concerns

1. Inadequate sample size: The limited cohort (n=18) of final-year nursing students from a single institution undermines statistical power and generalizability.

Response: Thank you for this insightful comment. We agree that the limited sample size and single-institution setting restrict the statistical power and generalizability of the findings. This study was designed as a pilot usability and feasibility evaluation, and the sample size (n=18) aligns with prior recommendations for usability studies (e.g., Lewis, 2021). Nevertheless, we acknowledge that broader validation is needed. To address this limitation, we have revised the Limitations section to explicitly state the constraints imposed by the small, homogeneous sample and to emphasize the need for future research with larger, more diverse cohorts across multiple institutions.

Change: “This study used a small sample size from a single institution, which may limit the statistical power and generalizability of the findings. Therefore, future research should recruit a larger and more diverse participant cohort from multiple institutions.” (Lines 381–383)

2. Absence of effectiveness evaluation: The study fails to assess educational outcomes (knowledge acquisition, skill transfer, or behavioral change) despite claiming the program is “effective."(describe)

Response: Thank you for this valuable feedback. We acknowledge that our study was designed as a pilot usability and feasibility evaluation, and therefore, we did not measure direct educational outcomes such as knowledge acquisition, skill performance, or behavioral change. We agree with the reviewer that using the term “effective” was misleading, as the data collected only support conclusions regarding usability, cybersickness, and presence. In response, we have revised the manuscript to avoid claims of “effectiveness” and instead describe the program as a “usable and immersive tool with potential for nursing education.” In the Abstract, Development, Discussion, and Conclusion sections, we have also clarified that future research is needed to evaluate educational outcomes to establish the program’s effectiveness.

Change:

• Abstract: “IVRsim-FMP demonstrated acceptable usability and high immersion, indicating potential as an educational tool for nursing education on fall prevention and management. This program provided opportunities to practice patient safety scenarios that are difficult to replicate in clinical environments. The findings suggest potential applications beyond nursing education, extending to continuous professional development for new and experienced nurses in fall prevention practices.” (Lines 21–26)

• Development of IVRsim-FMP – Evaluation Phase: “(4) Evaluation Phase

In the evaluation phase, 18 nursing students participated in a systematic usability assessment of the content. The evaluation measured system usability, cybersickness, and presence to determine the usability, feasibility, and potential applicability of IVRsim-FMP in nursing education.” (Lines 219–223)

• Discussion: “For digitally native nursing students, IVR simulation offers high levels of immersion and interactivity, making it a compelling educational tool. The findings of this study suggest that integrating IVR simulation into fall prevention education represents a promising pedagogical strategy.” (Lines 349–352)

• Conclusion: “The IVRsim-FMP program demonstrated acceptable usability, mild cybersickness, and high presence, indicating that it is a usable and immersive simulation tool with potential for nursing education in fall prevention and postfall patient care. By enabling practice in realistic patient safety scenarios that are difficult to replicate in authentic clinical settings, this program may also be adapted for ongoing fall prevention training among newly graduated and experienced nurses.

Future research can build on these findings by assessing the program’s impact on diverse learning outcomes and refining its realism and usability through user-centered design enhancements and additional immersive features. Such developments may extend the applicability of VR-based simulation to a wider range of contexts for patient safety education.” (Lines 392–401)

3. Omission of psycho-social dimensions: Critical psychological and social aspects of fall management are neglected in scenario design.

Response: Thank you for this feedback. We agree with the reviewer’s valuable comment that our fall management scenario primarily emphasized physical assessment and interventions, while important psychological and social aspects were not explicitly integrated. To address this point, we have revised the Discussion section to acknowledge this limitation and to highlight the importance of incorporating psycho-social dimensions in future iterations of the program. We have also cited recent studies that emphasize the role of VR in fostering empathy and understanding of patients’ and family members’ emotional needs, thereby strengthening the educational value of simulation.

Change: “VR has been demonstrated to facilitate learner development of greater empathy and understanding of patients’ emotional needs [42,43]. However, this study primarily focused on physical assessments and interventions for patients experiencing falls. Future research should enhance the program by incorporating emotional assessments and nursing interventions to support comprehensive patient-centered fall care. Moreover, integrating patient participation and emotional engagement strategies may further improve education on fall prevention. Furthermore, within the context of patient safety education, implementing error-reporting mechanisms in the virtual environment warrants consideration.” (Lines 317–324)

4. Deficient data analysis and discussion: Weak interpretation of results and insufficient bench-marking against existing literature diminish the study’s scholarly rigor.

Response: Thank you for this feedback. We sincerely appreciate the reviewer’s constructive comment regarding the need for a stronger interpretation of our results and benchmarking against prior literature. In response, we have substantially revised the Discussion section to address this concern. Specifically, we incorporated direct comparisons of our findings with previous VR-based nursing education studies, provided contextual explanations for the observed differences, and clarified the educational implications of our results.

Change:

• “The program achieved a high mean SUS score (M = 77.50, SD = 12.91), which falls within the ‘Good–Excellent’ usability range according to GitLab’s criteria [40]. Compared with prior nursing education studies using VR or simulation-based systems, such as Kim (M = 74.40) [11] and Yu et al. (M = 67.59) [12], the study’s result indicates relatively higher perceived usability. This elevated usability score may be explained by the program’s intuitive interface design, simplified navigation pathways, and clearly structured task sequences, which collectively reduced cognitive load and facilitated user engagement.” (Lines 331–337)

• “In addition, the program yielded high overall presence scores (M = 150.94), with particularly elevated scores in the ‘realism’ subdomain, which evaluates movement and immersion within the virtual space. This presence score was higher than that reported in a previous study that applied an IVR simulation to nursing students (M = 120.24) [44]. The higher presence observed in this study may be attributed to the integration of authentic clinical visuals, the inclusion of virtual EMR functions for reviewing diagnostic results, and the alignment of tasks with real-world nursing responsibilities, all of which enhanced ecological validity. Given that immersion in VR simulations has been linked to improved learning outcomes [45], future research should examine whether the high prese

# Peer review: Decision Letter 1

**DOI:** 10.1371/journal.pone.0357026.r003

**Authors:** Domna Banakou

24 Feb 2026

Dear Dr. Yun,

Thank you for submitting your manuscript to PLOS ONE. After careful consideration, we feel that it has merit but does not fully meet PLOS ONE’s publication criteria as it currently stands. Therefore, we invite you to submit a revised version of the manuscript that addresses the points raised during the review process.

Please submit your revised manuscript by Apr 10 2026 11:59PM. If you will need more time than this to complete your revisions, please reply to this message or contact the journal office at plosone@plos.org. When you're ready to submit your revision, log on to https://www.editorialmanager.com/pone/ and select the 'Submissions Needing Revision' folder to locate your manuscript file.

- A letter that responds to each point raised by the academic editor and reviewer(s). You should upload this letter as a separate file labeled 'Response to Reviewers'.
- A marked-up copy of your manuscript that highlights changes made to the original version. You should upload this as a separate file labeled 'Revised Manuscript with Track Changes'.
- An unmarked version of your revised paper without tracked changes. You should upload this as a separate file labeled 'Manuscript'.

If applicable, we recommend that you deposit your laboratory protocols in protocols.io to enhance the reproducibility of your results. Protocols.io assigns your protocol its own identifier (DOI) so that it can be cited independently in the future. For instructions see: https://journals.plos.org/plosone/s/submission-guidelines#loc-laboratory-protocols. Additionally, PLOS ONE offers an option for publishing peer-reviewed Lab Protocol articles, which describe protocols hosted on protocols.io. Read more information on sharing protocols at https://plos.org/protocols?utm_medium=editorial-email&utm_source=authorletters&utm_campaign=protocols.

We look forward to receiving your revised manuscript.

Kind regards,

Domna Banakou

Academic Editor

PLOS One

Journal Requirements:

If the reviewer comments include a recommendation to cite specific previously published works, please review and evaluate these publications to determine whether they are relevant and should be cited. There is no requirement to cite these works unless the editor has indicated otherwise.

Please review your reference list to ensure that it is complete and correct. If you have cited papers that have been retracted, please include the rationale for doing so in the manuscript text, or remove these references and replace them with relevant current references. Any changes to the reference list should be mentioned in the rebuttal letter that accompanies your revised manuscript. If you need to cite a retracted article, indicate the article’s retracted status in the References list and also include a citation and full reference for the retraction notice.

[Note: HTML markup is below. Please do not edit.]

Reviewers' comments:

Reviewer's Responses to Questions

**Comments to the Author**

Reviewer #2: All comments have been addressed

Reviewer #3: (No Response)

**********

2. Is the manuscript technically sound, and do the data support the conclusions?

Reviewer #2: Yes

Reviewer #3: Yes

**********

3. Has the statistical analysis been performed appropriately and rigorously?

Reviewer #2: Yes

Reviewer #3: Yes

**********

4. Have the authors made all data underlying the findings in their manuscript fully available?

The PLOS Data policy

Reviewer #2: Yes

Reviewer #3: Yes

**********

5. Is the manuscript presented in an intelligible fashion and written in standard English?

Reviewer #2: Yes

Reviewer #3: Yes

**********

Reviewer #2: Dear Authors,

Thank you for your attention and responses to the previous comments.

Please ensure consistency in the use of abbreviations throughout the text. Instances of inconsistent use of uppercase and lowercase letters, as well as repeated definition of the same abbreviation for a single term, were observed.

Thank you.

Reviewer #3: The general thesis of the paper is well formulated and the protocol and discussion is sound. However, there are some gaps that need addressing that I have included in my comments, which are attached. Most prominent among those is a deeper discussion about the absence of task performance metrics (on l.385) - is that data available? Even task completion times or success rates per participant would be a valuable metric of whether participants understood and were able to perform the task appropriately. It would also be valuable data for contrasting with the analogue version of the same nursing tasks and a justification of the claims the authors make around VR's superiority as a training tool. Please also include links to the figures, as I was unable to find them in the manuscript or reviewer portal.

**********

what does this mean?). If published, this will include your full peer review and any attached files.

If you choose “no”, your identity will remain anonymous but your review may still be made public.

**Do you want your identity to be public for this peer review?**  For information about this choice, including consent withdrawal, please see our Privacy Policy

Reviewer #2: No

Reviewer #3: No

**********

[NOTE: If reviewer comments were submitted as an attachment file, they will be attached to this email and accessible via the submission site. Please log into your account, locate the manuscript record, and check for the action link "View Attachments". If this link does not appear, there are no attachment files.]

To ensure your figures meet our technical requirements, please review our figure guidelines: https://journals.plos.org/plosone/s/figures

You may also use PLOS’s free figure tool, NAAS, to help you prepare publication quality figures: https://journals.plos.org/plosone/s/figures#loc-tools-for-figure-preparation.

NAAS will assess whether your figures meet our technical requirements by comparing each figure against our figure specifications.

# Author comment: Author response to Decision Letter 2

**DOI:** 10.1371/journal.pone.0357026.r004

24 Mar 2026

We thank the Academic Editor and Reviewers for their thoughtful and constructive comments. We have carefully revised the manuscript in response to all comments and have uploaded a separate detailed point-by-point response. In the revised manuscript, we improved the consistency of abbreviations and terminology throughout the text and tables, added simulation completion-time data as a preliminary objective indicator of task execution, expanded the Discussion regarding the absence of task performance metrics, clarified this issue further in the Limitations section, and rechecked all figure files to ensure that they are properly included and accessible in the revised submission. We believe that these revisions have strengthened the clarity and quality of the manuscript.

Attachment Submitted filename: Response to Reviewers_0310.docx

# Peer review: Decision Letter 2

**DOI:** 10.1371/journal.pone.0357026.r005

**Authors:** Domna Banakou

12 May 2026

Dear Dr. Yun,

Thank you for submitting your manuscript to PLOS ONE. After careful consideration, we feel that it has merit but does not fully meet PLOS ONE’s publication criteria as it currently stands. Therefore, we invite you to submit a revised version of the manuscript that addresses the points raised during the review process.

You should carefully revise the manuscript to improve precision and internal consistency. Please proofread for awkward or ungrammatical wording, check SUS reporting and provide full design details, including sample and pilot convenience sample. More broadly, the Discussion and Conclusion should be tightened so that claims remain aligned with the actual data, which support usability and immersion, but not knowledge gain, skill accuracy, retention, superiority over standard teaching, or broader patient-safety outcomes. Kindly refer to full details below.

Please submit your revised manuscript by Jun 26 2026 11:59PM. If you will need more time than this to complete your revisions, please reply to this message or contact the journal office at plosone@plos.org. When you're ready to submit your revision, log on to https://www.editorialmanager.com/pone/ and select the 'Submissions Needing Revision' folder to locate your manuscript file.

- A letter that responds to each point raised by the academic editor and reviewer(s). You should upload this letter as a separate file labeled 'Response to Reviewers'.
- A marked-up copy of your manuscript that highlights changes made to the original version. You should upload this as a separate file labeled 'Revised Manuscript with Track Changes'.
- An unmarked version of your revised paper without tracked changes. You should upload this as a separate file labeled 'Manuscript'.

If you would like to make changes to your financial disclosure, please include your updated statement in your cover letter. Guidelines for resubmitting your figure files are available below the reviewer comments at the end of this letter.

If applicable, we recommend that you deposit your laboratory protocols in protocols.io to enhance the reproducibility of your results. Protocols.io assigns your protocol its own identifier (DOI) so that it can be cited independently in the future. For instructions see: https://journals.plos.org/plosone/s/submission-guidelines#loc-laboratory-protocols. Additionally, PLOS ONE offers an option for publishing peer-reviewed Lab Protocol articles, which describe protocols hosted on protocols.io. Read more information on sharing protocols at https://plos.org/protocols?utm_medium=editorial-email&utm_source=authorletters&utm_campaign=protocols.

As the corresponding author, your ORCID iD is verified in the submission system and will appear in the published article. PLOS supports the use of ORCID, and we encourage all coauthors to register for an ORCID iD and use it as well. Please encourage your coauthors to verify their ORCID iD within the submission system before final acceptance, as unverified ORCID iDs will not appear in the published article. *Only*  the individual author can complete the verification step; PLOS staff *cannot*  verify ORCID iDs on behalf of authors.

We look forward to receiving your revised manuscript.

Kind regards,

Domna Banakou

Academic Editor

PLOS One

Journal Requirements:

If the reviewer comments include a recommendation to cite specific previously published works, please review and evaluate these publications to determine whether they are relevant and should be cited. There is no requirement to cite these works unless the editor has indicated otherwise.

Please review your reference list to ensure that it is complete and correct. If you have cited papers that have been retracted, please include the rationale for doing so in the manuscript text, or remove these references and replace them with relevant current references. Any changes to the reference list should be mentioned in the rebuttal letter that accompanies your revised manuscript. If you need to cite a retracted article, indicate the article’s retracted status in the References list and also include a citation and full reference for the retraction notice.

Additional Editor Comments (if provided):

There are multiple remaining issues that the authors should address:

1. The intro has a clear rationale but there are still a few weaknesses as some language is awkward or grammatically off (please proof read). The "digital natives" argument is dated and I would recommend either removing or softening the sentence, or update. Also, the novelty claim that no prior research has investigated VR for fall prevention education in nursing students should be phrased more cautiously, for example "to our knowledge". The term "usefulness" in the abstract is broader than what was actually measured and "usability and feasibility" would be more precise.

2. The SUS item reporting looks internally inconsistent. In Table 2, several negatively worded SUS items have very high means, for example: "I found the system unnecessarily complex" = 4.61, and

"I thought there was too much inconsistency in this system" = 4.50. With the stated 1–5 agreement scale, those raw means would imply users agreed the system was complex/inconsistent/cumbersome, which conflicts with the reported total SUS score of 77.50 and the conclusion that usability was acceptable. This needs correction or explanation. Please justify.

3. The manuscript defines "preliminary validity" using recruitment, protocol adherence, and data completion criteria from a pilot/feasibility paper. Those are really feasibility/process indicators, not validity in the psychometric or methodological sense. I would advise renaming this section to something like feasibility indicators or preliminary feasibility throughout the abstract, methods, and results.

4. Several design details are missing:

a. There are no participant characteristics related to prior VR experience, gaming familiarity, visual correction, or susceptibility to motion sickness, even though these are directly relevant to usability and cybersickness.

b. The sample-size rationale is lightweight and ends with recruitment stopping at 18 rather than the initially planned 20. That is not fatal, but it should be described more cleanly as a pilot convenience sample.

c. The methods say completion time was calculated among participants with valid time records, but the Results do not state how many participants had valid time data.

d. The paper says participants should stop if they experienced two or more symptoms, but it does not explicitly state whether any participants paused, stopped early, or needed assistance.

5. The descriptive results themselves are fine for a pilot usability paper, but a few parts need more details:

a. Table ranges appear to be observed ranges, not instrument ranges, and that should be labeled clearly.

b. The VRSQ reliability in this sample was low (a = 0.657), so conclusions about cybersickness should remain cautious.

c. Completion time should not be treated as evidence of adequate task performance.

d. The manuscript should avoid implying that high presence/usability means the program is educationally effective.

6. Your discussion sometimes slides from "usable and immersive" into claims that the program supports patient-safety skill acquisition, transfer to clinical contexts, and broader professional training. Those claims may be plausible, but this study did not measure knowledge gain, skill accuracy, retention, comparison with standard teaching, or patient-safety outcomes. The current limitations section partly corrects this, but the discussion and conclusion should be brought into tighter alignment with the actual data.

[Note: HTML markup is below. Please do not edit.]

Reviewers' comments:

Reviewer's Responses to Questions

**Comments to the Author**

Reviewer #3: (No Response)

**********

2. Is the manuscript technically sound, and do the data support the conclusions?

Reviewer #3: Yes

**********

3. Has the statistical analysis been performed appropriately and rigorously?

Reviewer #3: Yes

**********

4. Have the authors made all data underlying the findings in their manuscript fully available?

The PLOS Data policy

Reviewer #3: Yes

**********

5. Is the manuscript presented in an intelligible fashion and written in standard English?

Reviewer #3: Yes

**********

Reviewer #3: The revisions to the paper are definite improvements and my previous comments were appropriately addressed. However, I do have some additional (minor) comments that require attention before it can be accepted for publication.

line 290 Simulation completion time section: it would be beneficial to have a projected ideal completion time as a comparison point - how long is the fastest possible playthrough, for example (presumably done by one of the authors, who designed the system?) Some discussion of the ideal VR session time for a training simulation (also included in the literature review) would be appropriate in relation to the discussion of visual fatigue on l 379. It would also flesh out this new section, which is only a single sentence long.

line 395 - how many students encountered issues with their eye sight as a result of removing their glasses? It would be good to note the mean time without including the eyesight-impaired group, especially given its impact on the SSQ. In my experience headsets have spacers that allow users to accommodate wearing glasses with an HMD to overcome this issue.

line 398 and 409 - no need to repeat the very similar wording in adjacent sections, I would remove it from the discussion.

Once these are addressed, I see no further barriers to publication.

**********

what does this mean?). If published, this will include your full peer review and any attached files.

If you choose “no”, your identity will remain anonymous but your review may still be made public.

**Do you want your identity to be public for this peer review?**  For information about this choice, including consent withdrawal, please see our Privacy Policy

Reviewer #3: No

**********

[NOTE: If reviewer comments were submitted as an attachment file, they will be attached to this email and accessible via the submission site. Please log into your account, locate the manuscript record, and check for the action link "View Attachments". If this link does not appear, there are no attachment files.]

To ensure your figures meet our technical requirements, please review our figure guidelines: https://journals.plos.org/plosone/s/figures

You may also use PLOS’s free figure tool, NAAS, to help you prepare publication quality figures: https://journals.plos.org/plosone/s/figures#loc-tools-for-figure-preparation.

NAAS will assess whether your figures meet our technical requirements by comparing each figure against our figure specifications.

# Author comment: Author response to Decision Letter 3

**DOI:** 10.1371/journal.pone.0357026.r006

21 Jun 2026

Thank you for the opportunity to revise our manuscript. We have carefully addressed the editor’s and reviewer’s comments and have uploaded the revised manuscript, a tracked-changes version, and a response-to-reviewers file for your consideration.

Attachment Submitted filename: Reponse to Reviwers.docx

# Peer review: Decision Letter 3

**DOI:** 10.1371/journal.pone.0357026.r007

**Authors:** Domna Banakou

27 Jul 2026

Dear Dr. Yun,

Thank you for submitting your manuscript to PLOS ONE. After careful consideration, we feel that it has merit but does not fully meet PLOS ONE’s publication criteria as it currently stands. Therefore, we invite you to submit a revised version of the manuscript that addresses the points raised during the review process.

I would like to thank the author for the revisions. Kindly address some minor comments from the reviewers below. I would also recommend some final proof reading to fix some issues and improve overall quality of manuscript.

Please submit your revised manuscript by Sep 10 2026 11:59PM. If you will need more time than this to complete your revisions, please reply to this message or contact the journal office at plosone@plos.org. When you're ready to submit your revision, log on to https://www.editorialmanager.com/pone/ and select the 'Submissions Needing Revision' folder to locate your manuscript file.

- A letter that responds to each point raised by the academic editor and reviewer(s). You should upload this letter as a separate file labeled 'Response to Reviewers'.
- A marked-up copy of your manuscript that highlights changes made to the original version. You should upload this as a separate file labeled 'Revised Manuscript with Track Changes'.
- An unmarked version of your revised paper without tracked changes. You should upload this as a separate file labeled 'Manuscript'.

If you would like to make changes to your financial disclosure, please include your updated statement in your cover letter. Guidelines for resubmitting your figure files are available below the reviewer comments at the end of this letter.

If applicable, we recommend that you deposit your laboratory protocols in protocols.io to enhance the reproducibility of your results. Protocols.io assigns your protocol its own identifier (DOI) so that it can be cited independently in the future. For instructions see: https://journals.plos.org/plosone/s/submission-guidelines#loc-laboratory-protocols. Additionally, PLOS ONE offers an option for publishing peer-reviewed Lab Protocol articles, which describe protocols hosted on protocols.io. Read more information on sharing protocols at https://plos.org/protocols?utm_medium=editorial-email&utm_source=authorletters&utm_campaign=protocols.

As the corresponding author, your ORCID iD is verified in the submission system and will appear in the published article. PLOS supports the use of ORCID, and we encourage all coauthors to register for an ORCID iD and use it as well. Please encourage your coauthors to verify their ORCID iD within the submission system before final acceptance, as unverified ORCID iDs will not appear in the published article. *Only*  the individual author can complete the verification step; PLOS staff *cannot*  verify ORCID iDs on behalf of authors.

We look forward to receiving your revised manuscript.

Kind regards,

Domna Banakou

Academic Editor

PLOS One

Journal Requirements:

1. If the reviewer comments include a recommendation to cite specific previously published works, please review and evaluate these publications to determine whether they are relevant and should be cited. There is no requirement to cite these works unless the editor has indicated otherwise.

2. Please review your reference list to ensure that it is complete and correct. If you have cited papers that have been retracted, please include the rationale for doing so in the manuscript text, or remove these references and replace them with relevant current references. Any changes to the reference list should be mentioned in the rebuttal letter that accompanies your revised manuscript. If you need to cite a retracted article, indicate the article’s retracted status in the References list and also include a citation and full reference for the retraction notice.

[Note: HTML markup is below. Please do not edit.]

Reviewers' comments:

Reviewer's Responses to Questions

**Comments to the Author**

Reviewer #3: (No Response)

**********

2. Is the manuscript technically sound, and do the data support the conclusions?

Reviewer #3: Partly

**********

3. Has the statistical analysis been performed appropriately and rigorously?

Reviewer #3: No

**********

4. Have the authors made all data underlying the findings in their manuscript fully available?

The PLOS Data policy

Reviewer #3: Yes

**********

5. Is the manuscript presented in an intelligible fashion and written in standard English?

Reviewer #3: Yes

**********

Reviewer #3: I appreciate the revisions based on my previous comments. Now I just have some final concerns about the VRSQ calculations and labelling in table 3 and 4:

1. on l. 140 you say the system is based on a scale of 0-4 (Severe) - my understanding from Kim's paper is that the scale has 4 points, starting at 0, meaning the highest score is 3, not 4.

2. The paper calculates VRSQ-Total as a raw sum of the 9 items (max 36) but applies a scaled/weighted formula to VRSQ-O (max 50) and VRSQ-D (max 20), resulting in the Total score being lower than the Oculomotor subscale score (9.89 vs 14.89). Again, my understanding was that VRSQ-Total is calculated as the average of the two subscale percentages so that all three metrics sit on the exact same scale (0–100%).

3. Small detail, but the sub-scale's correct name in Table 4 should be Possibility to Examine, not Possibility to Examination.

One these are addressed we can hopefully proceed to publication.

**********

what does this mean?). If published, this will include your full peer review and any attached files.

If you choose “no”, your identity will remain anonymous but your review may still be made public.

**Do you want your identity to be public for this peer review?**  For information about this choice, including consent withdrawal, please see our Privacy Policy

Reviewer #3: No

**********

[NOTE: If reviewer comments were submitted as an attachment file, they will be attached to this email and accessible via the submission site. Please log into your account, locate the manuscript record, and check for the action link "View Attachments". If this link does not appear, there are no attachment files.]

To ensure your figures meet our technical requirements, please review our figure guidelines: https://journals.plos.org/plosone/s/figures

You may also use PLOS’s free figure tool, NAAS, to help you prepare publication quality figures: https://journals.plos.org/plosone/s/figures#loc-tools-for-figure-preparation.

NAAS will assess whether your figures meet our technical requirements by comparing each figure against our figure specifications.

# Author comment: Author response to Decision Letter 4

**DOI:** 10.1371/journal.pone.0357026.r008

10 Aug 2026

Thank you for the opportunity to revise our manuscript. We have carefully addressed the reviewer’s comments and revised the manuscript accordingly. Detailed responses and the corresponding revisions are provided in the uploaded “Response to Reviewers” file. The revised manuscript and related files have also been uploaded for your consideration.

Attachment Submitted filename: Reponse_to_Reviwers_auresp_4.docx

# Editor report: Decision Letter 4

**DOI:** 10.1371/journal.pone.0357026.r009

**Authors:** Domna Banakou

11 Aug 2026

Development of an immersive fall management program for nursing students: A usability study

PONE-D-25-23302R4

Dear Dr. Yun,

We’re pleased to inform you that your manuscript has been judged scientifically suitable for publication and will be formally accepted for publication once it meets all outstanding technical requirements.

Within one week, you’ll receive an e-mail detailing the required amendments. When these have been addressed, you’ll receive a formal acceptance letter and your manuscript will be scheduled for publication.

An invoice will be generated when your article is formally accepted. Please note, if your institution has a publishing partnership with PLOS and your article meets the relevant criteria, all or part of your publication costs will be covered. Please make sure your user information is up-to-date by logging into Editorial Manager at Editorial Manager® and clicking the ‘Update My Information' link at the top of the page. For questions related to billing, please contact billing support.

If your institution or institutions have a press office, please notify them about your upcoming paper to help maximize its impact. If they’ll be preparing press materials, please inform our press team as soon as possible -- no later than 48 hours after receiving the formal acceptance. Your manuscript will remain under strict press embargo until 2 pm Eastern Time on the date of publication. For more information, please contact onepress@plos.org.

Kind regards,

Domna Banakou

Academic Editor

PLOS One

# Editor report: Acceptance letter

**DOI:** 10.1371/journal.pone.0357026.r010

**Authors:** Domna Banakou

PONE-D-25-23302R4

PLOS One

Dear Dr. Yun,

I'm pleased to inform you that your manuscript has been deemed suitable for publication in PLOS One. Congratulations! Your manuscript is now being handed over to our production team.

At this stage, our production department will prepare your paper for publication. This includes ensuring the following:

* All references, tables, and figures are properly cited

* All relevant supporting information is included in the manuscript submission,

* There are no issues that prevent the paper from being properly typeset

You will receive further instructions from the production team, including instructions on how to review your proof when it is ready. Please keep in mind that we are working through a large volume of accepted articles, so please give us a few days to review your paper and let you know the next and final steps.

Lastly, if your institution or institutions have a press office, please let them know about your upcoming paper now to help maximize its impact. If they'll be preparing press materials, please inform our press team within the next 48 hours. Your manuscript will remain under strict press embargo until 2 pm Eastern Time on the date of publication. For more information, please contact onepress@plos.org.

You will receive an invoice from PLOS for your publication fee after your manuscript has reached the completed accept phase. If you receive an email requesting payment before acceptance or for any other service, this may be a phishing scheme. Learn how to identify phishing emails and protect your accounts at https://explore.plos.org/phishing.

If we can help with anything else, please email us at customercare@plos.org.

Thank you for submitting your work to PLOS One and supporting open access.

Kind regards,

PLOS ONE Editorial Office Staff

on behalf of

Dr. Domna Banakou

Academic Editor

PLOS One
